# The Age‐Dependent Resident Myonuclear Multi‐Omic Response to an Acute Skeletal Muscle Hypertrophic Stimulus in Mice

**DOI:** 10.1002/advs.202521633

**Published:** 2026-02-17

**Authors:** Pieter J. Koopmans, Ronald G. Jones, Ana Regina Cabrera, Francielly Morena, Nicholas P. Greene, John J. McCarthy, Ahmed Ismaeel, Yuan Wen, Kevin A. Murach

**Affiliations:** ^1^ Cell and Molecular Biology Graduate Program University of Arkansas Fayetteville Arkansas USA; ^2^ Molecular Muscle Mass Regulation (M3R) Laboratory, Department Health, Human Performance, & Recreation University of Arkansas Fayetteville Arkansas USA; ^3^ Cachexia Research Laboratory, Department Health, Human Performance, & Recreation University of Arkansas Fayetteville Arkansas USA; ^4^ Department of Physiology College of Medicine University of Kentucky Lexington Kentucky USA; ^5^ Center For Muscle Biology College of Health Sciences University of Kentucky Lexington Kentucky USA; ^6^ Department of Anatomy, Physiology, and Pharmacology, College of Medicine Auburn University Auburn Alabama USA; ^7^ Division of Biomedical Informatics Department of Internal Medicine College of Medicine University of Kentucky Lexington Kentucky USA

**Keywords:** aging, overload, RNA‐seq, RRBS, smnRNA‐seq

## Abstract

A detailed analysis of how muscle fiber nuclei (myonuclei) respond to a hypertrophic stimulus could provide a critical step toward understanding compromised skeletal muscle plasticity with age. We used recombination‐independent doxycycline‐inducible myonucleus‐specific fluorescent labelling, tissue RNA‐sequencing, myonuclear DNA methylation analysis, multi‐omic integration, and single myonucleus RNA‐sequencing (smnRNA‐seq) to define the molecular characteristics of adult (6–8 month) and aged (24 month) murine skeletal muscle after acute mechanical overload (MOV). In adult and aged MOV muscles, we found that: 1) similarities in the transcriptional response to loading—specifically in metabolism genes – were partly explained by a post‐transcriptional microRNA‐mediated mechanism that we corroborated using an inducible muscle fiber‐specific *miR‐1* knockout model, 2) differences in age‐dependent transcriptional responses were linked to the magnitude and location of differential DNA methylation in resident myonuclei, specifically around genes such as *Myc*, *Runx1*, *Mybph*, *Ankrd1*, collagen (*Col*) genes, and minichromosome maintenance (*Mcm*) genes, 3) adult and aged resident myonuclear transcriptomes had differing enrichment for innervation‐related transcripts as well as unique transcriptional profiles in an *Atf3+* “sarcomere assembly” population after MOV, and 4) cellular deconvolution analysis and smnRNA‐seq supports a role for neuromuscular junction regulation in age‐specific hypertrophic adaptation. These data are a roadmap for uncovering molecular targets to enhance aged muscle adaptability.

## Graphical abstract

Resident myonuclei are the molecular “control centers” for large multinuclear muscle fibers. It is presumed that, with aging, these control centers become compromised and contribute to delayed or blunted muscle adaptive potential. This study is a detailed roadmap that exposes how young versus aged myonuclei respond to a hypertrophic loading stimulus across several molecular layers and at single myonucleus resolution.

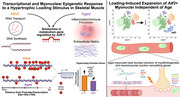

## 1 Introduction

Resistance exercise training is recognized as the most effective tool to induce muscle growth in humans [1, 2, 3]. Resistance training is also the most successful and accessible strategy to attenuate the natural and inevitable loss of skeletal muscle mass and function that ensues throughout the lifespan, termed age‐related sarcopenia [1, 4, 5, 6]. Unfortunately, there is evidence to suggest that the efficacy of resistance training tends to be impaired in aged compared to younger adult human populations [7, 8, 9]. In our hands, this observation is corroborated by murine models of muscle hypertrophy such as synergist ablation‐induced mechanical overload (MOV) and progressive weighted wheel running, where aged mice have attenuated hypertrophic outcomes compared to young [10, 11].

One barrier to pinpointing the molecular mechanism(s) for an age‐dependent reduction of muscle plasticity is the syncytial nature of skeletal muscle fibers (myofibers). Myofibers are massive cells that contain hundreds to thousands of individual and often specialized nuclei (myonuclei). Beyond the contractile myofibers, skeletal muscle comprises a heterogenous cellular milieu including numerous mononuclear cell types such as immune cells, fibroadipogenic progenitors (FAPs), fusogenic muscle stem cells (satellite cells), and endothelial cells, among others. In whole tissue, the molecular profiles of these supporting cell types can disguise processes occurring specifically within myofibers, which is the cell type that physically grows in response to mechanical stimuli. Furthermore, individual myonuclei within a given muscle fiber, or within a fiber of a specific myosin heavy chain (MyHC) type (e.g. slow versus fast) may display unique impairments with aging. Manually isolating individual muscle fibers to interrogate molecular contributors to muscle plasticity is tedious and low‐throughput and does not capture the complexity of individual myonuclear responses. Manual isolation of fibers can also be subject to contamination by adherent mononuclear cell types; this is especially true during times of stress characterized by mononuclear cell infiltration and a dramatic shift in muscle nuclear proportion away from myonuclei [12]. For these reasons, how myonuclei regulate muscle adaptation at the molecular level across the lifespan remains poorly understood. This lack of detailed information is a significant gap in the skeletal muscle aging literature [13, 14].

**FIGURE 1:**
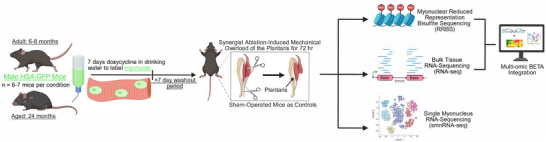
Schematic overview of experimental design.

To overcome the technical barriers of assessing muscle‐fiber specific molecular profiles, we used a genetically modified doxycycline‐inducible mouse model called HSA‐GFP (HSA = human skeletal actin promoter; GFP = green fluorescent protein) [12, 15]. This model is a recombination‐independent tool that allows for fluorescent myonuclear labeling in vivo and sorting‐based isolation of myonuclei with high specificity. Carefully timed myonuclear labeling with the HSA‐GFP model eliminates the possibility of capturing nuclei from other cell types as well as myonuclei derived from newly fused satellite cells. This labeling and purification approach allows us to focus specifically on resident myonuclei, which initiate and drive the muscle fiber hypertrophic process [14]. Using this tool, we sought to define the resident myonuclear molecular response to a known hypertrophic stimulus (MOV) in mice according to age: 6–8‐month adult versus 24‐month aged. MOV shares many of the same molecular responses to acute resistance exercise in humans, and like human resistance training, has lower hypertrophic efficacy with aging [9, 16, 17, 18]. We combined bulk tissue RNA‐sequencing, myonucleus specific DNA methylation, ‐omic integration, computational cellular deconvolution, and single myonucleus RNA‐sequencing (smnRNA‐seq) to provide a detailed portrait of how the early phase of loading‐induced muscle growth is regulated by resident myonuclei (Figure 1). These data serve as a resource to the skeletal muscle and aging research communities and provide fundamental molecular insight into how muscle plasticity can be affected late in life.

## 2 Results

### 2.1 The Effect of Age on the Skeletal Muscle Transcriptome in Sedentary Mice

**FIGURE 2:**
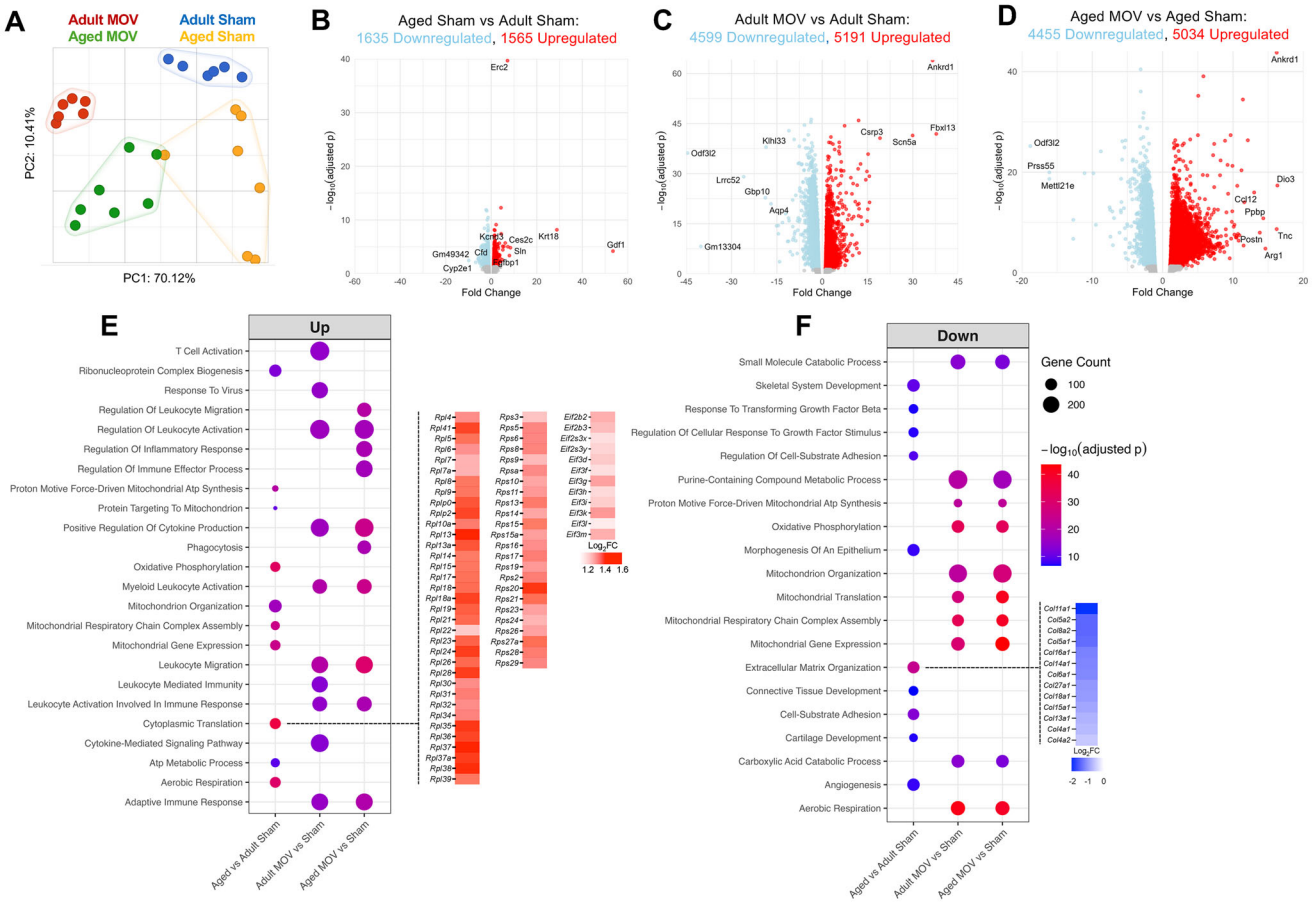
Tissue homogenate RNA‐sequencing in adult and aged sham and mechanical overload (MOV) skeletal muscle. (A) Principal component analysis of top 2000 features across experimental conditions. Volcano plots of (B) Aged sham vs adult sham, (C) Adult MOV vs adult sham, (D) Aged MOV vs aged sham (adj. *p*‐value < 0.05). (E) Plot showing top 10 GO biological processes pathways based on up‐regulated genes from each comparison. (F) Plot showing top 10 GO biological processes pathways based on down‐regulated genes from each comparison. MOV = mechanical overload. FC = fold‐change. Sample sizes were n = 6‐7, differentially expressed genes were calculated by DESeq2 with Benjamini–Hochberg correction method, and are significant at adj. p<0.05.

To assess baseline transcriptome differences in muscle with aging, we compared whole muscle gene expression using bulk RNA‐sequencing data from plantaris muscles between adult (6–8‐month, n = 6) and aged (24‐month, n = 7) male sham surgery control mice (File S1). Principal component analysis (PCA) revealed distinct differences between the transcriptomes of adult and aged skeletal muscle (Figure 2A). Notably, there is greater dispersion of data in aged sham versus adult sham, indicative of more transcriptional variability and/or stochasticity when aged, in agreement with prior work [19]. We identified 3,200 differentially expressed protein‐coding genes (DEGs) between aged sham versus adult sham mice (1,565 upregulated; 1,635 downregulated, adj. *p*<0.05, Figure 2B). Background corrected gene set enrichment analyses (Biological Processes) were performed separately on up‐ and down‐regulated genes [20, 21]. With age, upregulated gene ontology (GO) terms were primarily associated with mitochondrial gene expression, translation, and ribosome structural constituent genes (Figure 2E). These pathways were defined by numerous ribosomal protein genes (*Rps and Rpl)* and eukaryotic initiation factor (*Eif*) subunits. Although seemingly counterintuitive, our observation of upregulated ribosomal regulation in aged mouse muscle is consistent with prior work showing ribosomal RNA and proteins are elevated in aged human muscle at rest [22]. Furthermore, it is becoming clear that hyperactive mTORC1 signaling and elevated protein synthesis with aging is a driver of impaired proteostasis and sarcopenia, and that partial inhibition of mTORC1 with rapamycin reverses age‐related muscle deficits in sedentary animals [23, 24, 25, 26, 27]. Elevated levels of translation‐related genes with age tracks with these findings. Downregulated GO terms were related to skeletal system development, response to growth factors, and angiogenesis, but terms related to extracellular matrix (ECM) organization were most dominant (Figure 2F). Downregulation of these gene classes with aging may signal a loss of cellular identity accompanied by impaired ECM remodeling; the latter is established as a characteristic of aging skeletal muscle [28, 29, 30, 31, 32].

### 2.2 Conservation of Inflammation‐Oriented and Metabolic Transcriptional Profiles Between Adult and Aged Mouse Muscle Tissue Homogenate in Response to an Acute Hypertrophic Stimulus

We next sought to characterize the transcriptome of skeletal muscle (plantaris) in response to acute (72‐h) synergist ablation‐induced MOV in adult and aged male mouse muscle tissue (File S1). This early timepoint precedes bona fide muscle fiber growth but is determinant for the eventual myofibrillar muscle growth response [14, 33]. PCA revealed greater variability in aged versus adult animals in response to MOV (Figure 2A). In adult mice, 9,700 DEGs were identified in MOV versus sham (5,191 upregulated, 4,599 downregulated, adj. *p*<0.05, Figure 2C). In aged mice, 9,489 DEGs were identified in MOV versus sham (5,034 upregulated, 4,455 downregulated, adj. *p* < 0.05, Figure 2D). GO pathway analyses (top 10 biological processes shown) from DEGs in adult and aged MOV conditions, respectively, showed generally similar enriched GO terms. Upregulated GO pathways in adult and aged were overwhelmingly related to immune responses (Figure 2E). Induction of an immune response at the tissue level in muscle is unsurprising given pronounced inflammatory cell appearance at this early stage of MOV [12]. Downregulated GO terms were dominated by metabolic‐related processes, including mitochondrial organization, cellular respiration, and energy metabolism (Figure 2F). The signature of downregulated mitochondrial‐related genes may be a sign of the Warburg effect, like what is observed in cancer cells during rapid growth [34, 35]. We previously demonstrated this effect to occur during rapid MOV‐induced muscle hypertrophy of the plantaris muscle in mice [36]. This metabolic response to MOV results in a biasing of substrates toward “aerobic glycolysis” and the pentose phosphate pathway to support biomass accumulation (nucleotide synthesis), concomitant with reduced mitochondrial respiration [36, 37]. Evidence for the metabolic biasing of substrates has been shown in muscle growth contexts such as resistance training in humans [34, 38]. Our data suggest that a Warburg‐like metabolic signature appears to be conserved with MOV across ages during MOV.

### 2.3 Regulation of Golgi‐Related and Mitochondrial Gene Expression by miR‐1 Independent of Age

**FIGURE 3:**
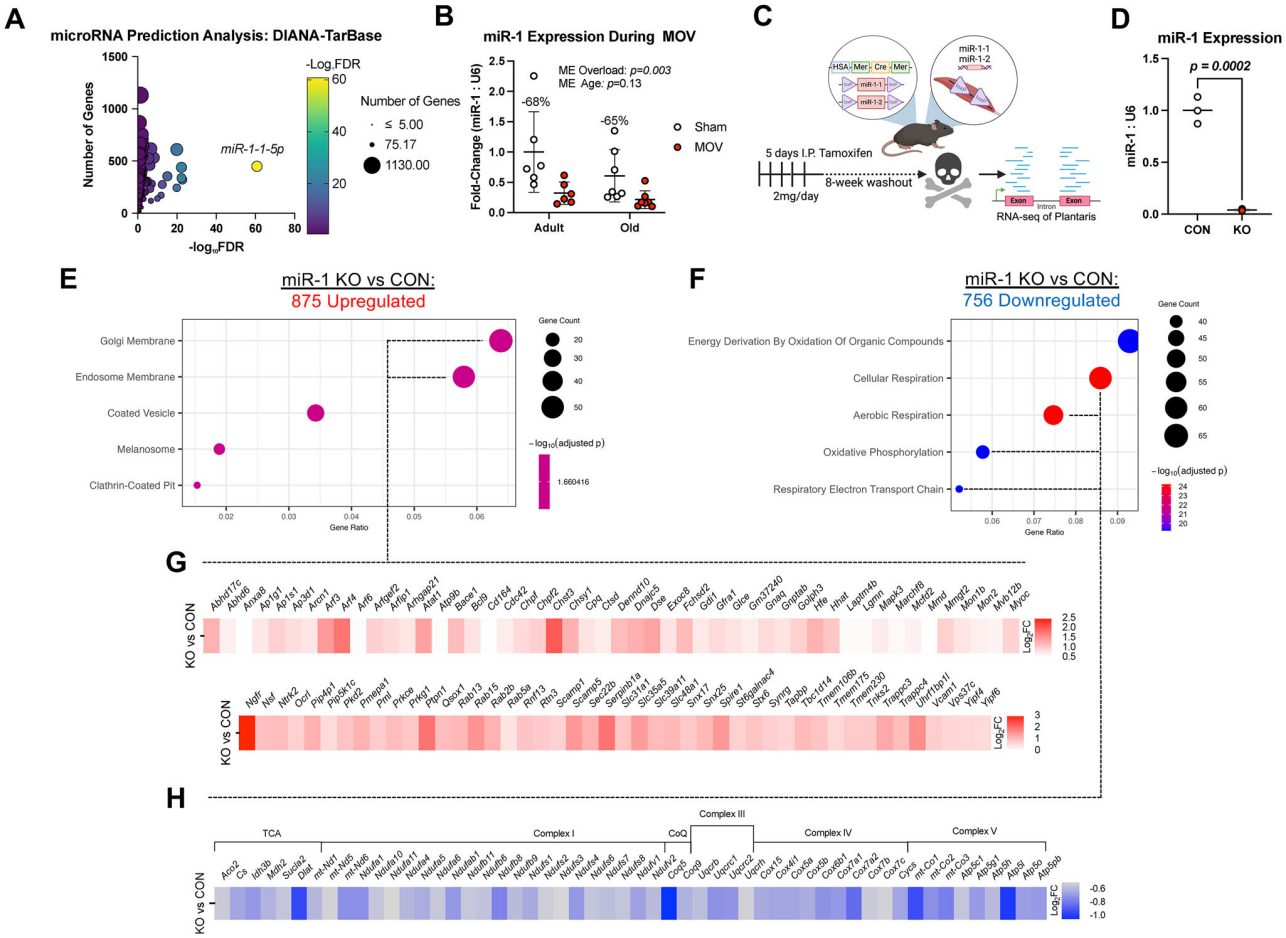
Evidence that *miR‐1* downregulation contributes to muscle transcriptome similarities between adult and aged MOV responses in tissue homogenate. (A) DIANA‐TarBase microRNA regulation prediction analysis from upregulated genes in response to MOV in both age conditions. (B) RT‐qPCR of *myomiR‐1* expression following MOV in adult and aged mice. (C) Experimental design summary of tamoxifen inducible muscle‐specific *miR‐1* knockout model (HSA‐miR‐1) and RNA‐sequencing analysis. (D) RT‐qPCR of *miR‐1* expression with tamoxifen treatment in HSA‐miR‐1 mouse model. (E) GO Cellular Components from genes upregulated by *miR‐1* KO. (F) GO Biological Processes from genes downregulated by *miR‐1* KO. (G) Heatmap of upregulated endomembrane and Golgi membrane system‐related genes. (H) Heatmap of downregulated TCA cycle and ETC‐related genes, MOV = Synergist‐Ablation Induced Mechanical Overload. ME = main effect. CON = control. KO = knock out. TCA = tricarboxylic acid cycle. CoQ = Coenzyme Q. Variables are presented at mean ± SD. For MOV experiment, sample sizes are n = 6–7 and data were compared by two‐way ANOVA test followed by a Tukey post‐hoc test across groups. For *miR‐1* KO experiment, sample sizes are n = 3 and independent two‐tailed t‐tests were performed. For RNA‐Sequencing data, differentially expressed genes were calculated by DESeq2 with Benjamini–Hochberg correction method. In all cases, significance was defined as p or adj. p ≤ 0.05. Statistical analysis was carried out using GraphPad Prism version 10.6.

Our recent work suggests that miRNAs are powerful regulators of the skeletal muscle transcriptome and the metabolic response to MOV [36, 37] as well as exercise training responses in murine skeletal muscle [39]. Given our recent findings, we used DIANA‐TarBase, an experimentally validated miRNA:gene expression database [40], to guide our analysis of influential miRNAs affecting gene expression with MOV independent of age. The microRNA predicted to be most explanatory for gene upregulation with MOV regardless of age was the myomiR *miR‐1* (Figure 3A). *miR‐1* is a muscle‐enriched microRNA hypothesized to act as a “molecular brake” on muscle growth given its downregulation coincides with hypertrophic stimuli in murine and human muscle [37, 39, 41, 42, 43].

In the present study, *miR‐1* levels were precipitously lower with MOV regardless of age (Figure 3B). To determine how *miR‐1* influences muscle gene expression in the plantaris muscle, we used a skeletal muscle fiber‐specific Cre‐mediated tamoxifen‐inducible *miR‐1* knockout (KO) mouse model, HSA‐miR‐1 (Figure 3C, Figure S1A and File S2) [41]. This mouse allows for simultaneous muscle‐specific inducible deletion of *miR‐1‐1* and *miR‐1‐2* (referred to as *miR‐1*) in adult muscle. Using middle‐aged experimental mice (12‐14 months at euthanasia), *miR‐1* was depleted in the plantaris by >95% relative to tamoxifen‐treated controls (Figure 3D). RNA‐seq revealed 875 upregulated and 756 downregulated genes after *miR‐1* depletion (Figures 3E,F). Gene set enrichment analyses indicated upregulation of Golgi membrane and endomembrane‐related genes (Figure 3E) with *miR‐1* depletion. This expression profile aligns with a previous report on *miR‐1* overexpression in skeletal muscle during MOV, where *miR‐1* induction blunted hypertrophy (Figure 3E,G) [43]. In our *miR‐1* KO RNA‐seq, ADP‐ribosylation factor 4 (*Arf4)*, which regulates endosome recycling and intracellular trafficking [44, 45], is a validated Argonaute 2 enhanced crosslinking and immunoprecipitation sequencing (AGO2 eCLIP‐seq) target of miR‐1 in human muscle tissue [41], as are cell‐cycle regulators that are implicated in tumorigenesis: *Azin1* and *Ptpn1* [46, 47, 48] (Figure S1). Genes related to cellular respiration and oxidoreductase activity were strongly downregulated in *miR‐1* KO relative to controls (Figures 3F,H). There was appreciable overlap in the transcriptomes between *miR‐1* KO and MOV, specifically related to downregulation of mitochondrial enzymes and mitoribosome‐related genes (Figure S1B). *miR‐1* was previously shown to control glycolytic flux and mitochondrial respiration in the oxidative soleus muscle of adult mice [41]. We comprehensively compared AGO2 eCLIP‐defined *miR‐1* targets in human skeletal muscle [41] to DEGs with MOV in adult and aged. We found an ∼80% overlap in the number of DEGs between adult and aged (File S1). Of those, few had marked differences in fold changes between adult and aged MOV. Altogether, our data indicate *miR‐1* is likely responsible for controlling the metabolic response as well as Golgi membrane remodeling processes during MOV in the fast‐twitch plantaris muscle irrespective of age. A *miR‐1* mediated post‐transcriptionally controlled metabolic switch may help to create a permissive environment for muscle growth [43].

### 2.4 Computational Deconvolution of Cell Type Contributions to MOV in Adult versus Aged Muscle Points to Differences at the Neuromuscular Junction

**FIGURE 4:**
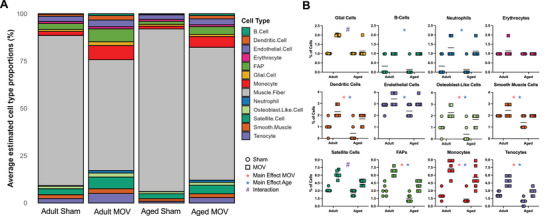
Inferring cell type proportions in adult versus aged MOV using bulk RNA‐sequencing and a published single cell RNA‐seq dataset. (A) Digital deconvolution of cell proportion from MOV bulk RNA‐sequencing data using Granulator and Zhang et al., *Cell Reports*, 2024^51^ as a reference scRNA‐seq dataset to delineate contributions to the global transcriptome. (B) Predicted proportion of cell types. Main effect of MOV = red asterisk. Main effect of Age = blue asterisk. Interaction = #. Continuous variables are presented as mean ± SD, with sample sizes of n = 6–7. Data were compared by two‐way ANOVA test followed by a Tukey post‐hoc test across groups. Significance was defined as p ≤ 0.05. Statistical analysis was carried out using GraphPad Prism version 10.6.

We previously performed deconvolution analysis of bulk RNA‐sequencing data using a reference single cell RNA‐seq (scRNA‐seq) muscle regeneration dataset to infer which cell types contributed to the global muscle MOV transcriptome in young mice (∼2 months old) [49, 50]. To determine if predicted changes in cell proportion during MOV is influenced by age, we performed cellular deconvolution using a recent (and more well‐suited) scRNA‐seq dataset from a 4‐day plantaris tenotomy MOV experiment (Figure 4A) [51]. In both adult and aged MOV, myonuclei (which typically appear in single cell datasets) were the major source of transcription. Fibro‐adipogenic progenitors (FAPs) and monocytes were the next largest contributors. Statistical analyses of predicted cellular proportions suggested greater abundance of glial cell and muscle satellite cell populations in adult versus aged mice during MOV (Figure 4B). In aged muscle, satellite cell activation and proliferation is impaired during muscle hypertrophy, so the present result is not unexpected [52, 53, 54]. Glial cells promote neuromuscular junction integrity during denervation and express genes implicated in the ECM [54]. Glial cells may also coordinate with satellite cells to support neuromuscular junction repair during muscle injury [55]. The precise role of glial cells during MOV is not well‐defined but could be related to neuromuscular junction remodeling. Recent evidence from our laboratory suggests subsets of aged satellite cells are enriched for neuromuscular genes during MOV, potentially contributing to innervation [56]. We previously showed how depletion of satellite cells in adult muscle results in excessive collagen accumulation concomitant with blunted long‐term muscle growth [33, 57, 58]. Collectively, our analysis suggests satellite cell, glial cell, and innervation‐related processes may be compromised during MOV with aging, which could in part explain age‐dependent muscle plasticity.

### 2.5 Distinctions in Age‐related Gene Expression in Muscle Tissue Homogenate During MOV

**FIGURE 5:**
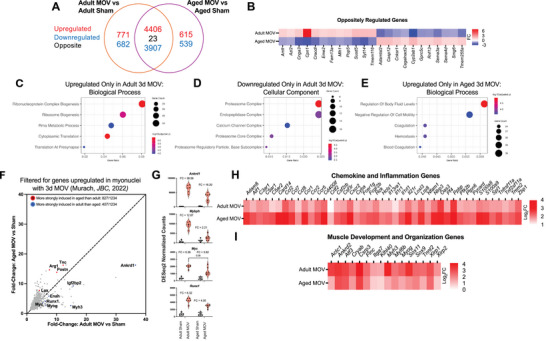
The adult versus aged differential transcriptome response to MOV in tissue homogenate and comparison to prior MOV myonucleus RNA‐sequencing data in young mice. (A) Venn diagram comparing overlap in differentially expressed genes in adult and aged conditions in response to MOV. (B) Heatmap with fold changes of genes that are oppositely regulated, upregulated in adult MOV but downregulated in aged MOV, downregulated in adult MOV but upregulated in aged MOV. (C) Top 5 enriched GO Biological Processes from upregulated DEGs exclusive to adult MOV, (D) Top 5 enriched GO Biological Processes from downregulated DEGs exclusive to adult MOV. (E) Top 5 enriched GO Biological Processes from upregulated DEGs exclusive to aged MOV. (F) Fold‐change induction of genes filtered for expression in myonuclei (Murach et al. *Journal of Biological Chemistry*, 2022^49^), (G) Violin plots showing normalized counts and fold changes of select myonuclei enriched genes (Aged MOV vs Adult MOV adj. *p*‐values: *Ankrd1*: <0.01; *Mybph*: <0.0001; *Myc*: 0.09; *Runx1*: 0.12), (H) Heatmap of Chemokine and Inflammation Genes. (I) Heatmap of Muscle Development and Organization‐Related Genes. FC = fold‐change. Sample sizes were n = 6‐7, differentially expressed genes were calculated by DESeq2 with Benjamini–Hochberg correction method, and are significant at adj. p<0.05. Continuous variables are presented as mean ± SD, with sample sizes of n = 6‐7, and were compared by two‐way ANOVA test followed by a Tukey post‐hoc test across groups.

Broad transcriptional patterns at the tissue level were generally conserved between adult and aged mice with MOV (Figures 2 and  5A); however, there were still numerous noteworthy distinctions. The overall transcriptional response to acute MOV was profound regardless of age, so despite significant overlap in DEGs (>8,300 shared between adult and aged MOV), there remained >1,400 DEGs exclusive to adult mice and >1,100 DEGs exclusive to aged mice (adj. *p*<0.05 relative to respective controls, Figure 5A). These genes may be important for explaining compromised muscle plasticity when aged.

Twenty‐three genes were oppositely regulated in adult versus aged mice after MOV (adj. *p*<0.05). Figure 5B shows genes that were upregulated in adult and downregulated in aged or vice versa. One such gene is *Casp12*, which was downregulated with MOV in adult and upregulated in aged. *Casp12* is an initiator of caspase leading to apoptosis. Deletion of *Casp12* preserves muscle function and reduces signs of muscle degeneration in *mdx* mice [59]; how this gene functions during MOV is not yet clear. *Sema3a* was also downregulated with MOV in adult but upregulated in aged MOV. *Sema3* is implicated in muscle mass regulation, where overexpression in IIB muscle fibers – a major myosin heavy chain isoform in plantaris muscle – prevents fusion of *Tw2+* myogenic progenitor cells (MPCs) to the muscle fiber [60]. Genetic ablation of *Tw2+* MPCs causes IIB fiber atrophy [61]. Perhaps the upregulation of *Sema3a* in aged mice during MOV prevents fusion of this IIB‐specific myogenic progenitor population and affects muscle adaptation. Importantly, *Twist2*+ cells may also be found in human skeletal muscle and are reportedly responsive to aging and resistance exercise [62, 63]. Enriched GO biological process terms from upregulated DEGs exclusive to adult MOV were related to ribonucleoprotein complex biogenesis, ribosome biogenesis, and translation at the pre‐synapse (Figure 5C). Enriched GO terms from downregulated DEGs exclusive to adult MOV (Figure 5D) were related to the proteasome and endopeptidase complex. By contrast, enriched GO terms from upregulated DEGs exclusive to aged MOV mice were related to coagulation, hemostasis, and negative regulation of cell motility. The unique upregulated genes in aged MOV collectively point to wound repair processes (Figure 5E) and suggest an altered early response to MOV when aged. There were no enriched GO terms for downregulated DEGs exclusive to aged MOV.

To further refine our examination of the response to MOV, we focused on genes previously identified to be enriched specifically in bulk myonuclei and upregulated in response to 72‐h MOV in young mice (1,694 genes) [49] and compared these with our bulk RNA‐seq in young and aged. There were 1,234 myonuclear MOV DEGs overlapping with the tissue RNA‐seq herein, of which 827 were more strongly induced in aged muscle than adult, and 407 more strongly induced in adult muscle than aged (Figure 5F). *Arg1, Lox, Tnc*, and *Postn*, and *Timp1* were more strongly induced in aged muscle than adult (Figure 5F). Differential regulation of these genes may relate to dysregulated ECM remodeling with MOV in aged. *Arg1* and *Timp1* expression is not exclusive to muscle fibers. *Arg1* is also upregulated in macrophages after 4 days of MOV [64], and *Timp1* is a macrophage‐derived pro‐inflammatory cytokine [65, 66, 67]. Several genes we previously identified to be enriched in myonuclei and associated with muscle mass regulation had a larger magnitude of induction in adult versus aged muscle, including *Myc, Runx1, Mybph*, and *Ankrd1* (Figure 5G). We recently identified skeletal muscle‐specific induction of *Myc*, a highly exercise‐responsive Yamanaka transcription factor and oncogene, to be sufficient for muscle growth [68]. *Myc* is shown to be less responsive to a muscle growth stimulus with age across species and conditions [69, 70, 71, 72, 73]; perhaps this age‐associated attenuation contributes to impaired muscle adaptive potential [49, 69, 71, 72] and may explain aging‐associated declines in loading‐mediated ribosome biogenesis [9]. *Runx1*, while induced strongly during MOV, is also upregulated during muscle denervation. *Runx1* may act as a pro‐hypertrophy signal as well as an anti‐atrophy countermeasure given depletion of *Runx1* decreases ribosome biogenesis in hematopoietic stem cells and exacerbates muscle wasting and myofibrillar disorganization during denervation [74, 75, 76]. Chemokine and inflammation‐oriented genes were more strongly induced in aged mice with MOV relative to adult (Figure 5H). Genes related to muscle development and organization were more strongly induced in adult muscle (Figure 5I). The stronger induction of muscle development and organization genes at the tissue‐level with MOV when younger could be related to a more robust satellite cell response versus old. We recently provided evidence for the presence of satellite cells epigenetically reinforcing muscle identity genes during lifelong wheel running [77]. Overall, distinctions in the bulk transcriptome of adult versus aged mouse muscle could contribute to reduced plasticity with loading when aged.

### 2.6 The Myonuclear DNA Methylome Is Significantly Altered by Age and Is Considerably Less Responsive to MOV in Aged Than Adult Muscle

**FIGURE 6:**
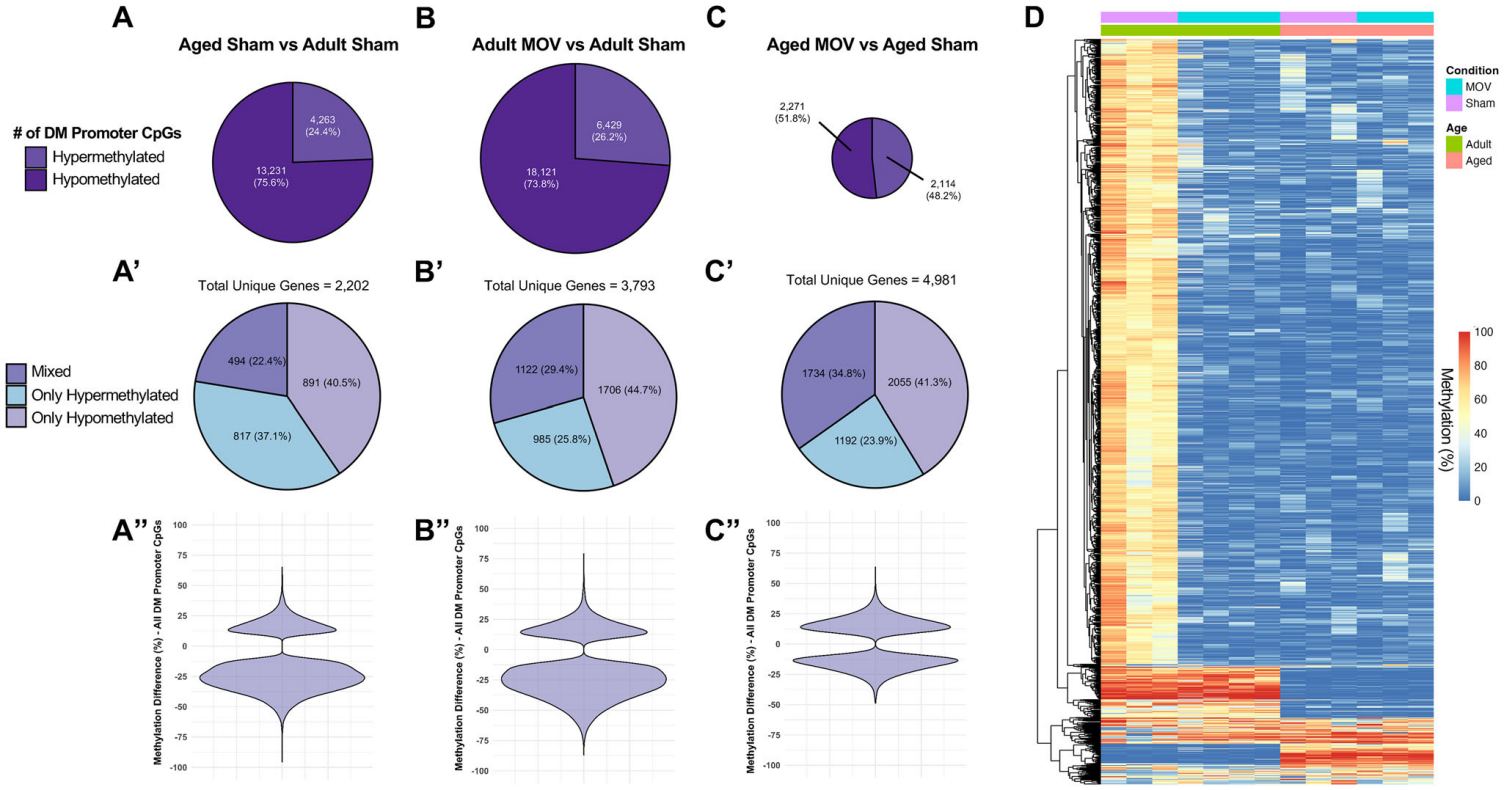
The myonuclear DNA methylome in adult and aged sham and MOV muscles. Number of promoter CpG sites with differential methylation for: (A) aged sham vs adult sham, (B) adult MOV vs adult sham, (C) aged MOV vs aged sham. Number of unique genes which are of mixed methylation status, only hypomethylated, or only hypermethylated for: (A’) aged sham vs adult sham, (B’) adult MOV vs adult sham, (C’) aged MOV vs aged sham. Violin plots of % differential methylation for: (A”) aged sham vs adult sham, (B”) adult MOV vs adult sham, (C”) aged MOV vs aged sham. (D) Hierarchical clustering heatmap of top 2000 variable DM CpG sites. DM = differentially methylated. Differentially methylated sites were defined as q‐value of <0.05 and >10% methylation difference. Samples sizes for each condition of: adult sham (n = 3), adult MOV (n = 4), aged sham (n = 3), aged MOV (n = 3). with Benjamini–Hochberg correction method and are significant at adj. p<0.05.

Using reduced representation bisulfite sequencing (RRBS), we previously reported the myonuclear DNA methylome is altered by acute MOV in young female mice (∼2 months old) [12, 36]. However, to date, the myonuclear DNA methylome response to MOV has not been defined in adult or aged muscle. We performed low‐input RRBS on myonuclei isolated via fluorescence activated nuclear sorting (FANS) from the plantaris muscle of sham control and MOV mice. We initially focused on promoter CpG methylation as this is canonically linked to gene expression regulation when compared with other genomic contexts such as exons/introns [78, 79]. Examining the effect of age (aged sham versus adult sham), 17,494 promoter CpG sites across 2,202 unique genes were differentially methylated (DM, Figure 6A&A′, adj. *p*<0.05 and >10% methylation difference). Around 22% of age‐related DM genes were both hypo‐ and hypermethylated (“mixed”), with the other 78% of genes being exclusively hypo‐ or hypermethylated. Many DM CpG sites were altered by up to ±25%–50% (Figure 6A″), indicating the myonuclear DNA methylome is dramatically altered by age alone, consistent with reports in muscle tissue of mice and humans [80, 81, 82, 83].

In adult MOV, there were 24,550 DM promoter CpGs relative to age‐matched sham, whereas in aged MOV there were only 4,385 DM CpGs (Figures 6B,C). This striking difference suggests the myonuclear methylome is less flexible after short‐term MOV in aged myonuclei. A lack of myonuclear epigenetic flexibility is perhaps due in part to the alterations with age observed in resting conditions (see above). For MOV comparisons, the DM CpGs corresponded to 3,793 unique genes in adult and 4,981 unique genes in aged, respectively (Figure 6B,C). Aged muscle had fewer differentially methylated sites but in more genes with MOV, suggesting a more uncoordinated epigenetic response to loading versus adult. Less epigenetic flexibility in aged muscle is further evidenced by a relatively lower percent change in methylation of individual CpG sites compared to adult (Figure 6B,C). In aged MOV, most CpG sites exhibited ±10%–25% differential methylation relative to control myonuclei with few outside of that range (maximum of ±50%). In contrast, adult MOV had more CpG sites altered by ≥25% and up to ±80% methylation difference. The marked differences in the magnitude of DNA methylation responses to MOV between adult and aged mice are illustrated by the heatmap of the top 2000 most variable CpG sites in Figure 6D. It is worth noting that with MOV in adult and aged, 65%–71% of altered genes that had differential methylation in promoter regions were only hypo‐ or hypermethylated, whereas 29%–35% of genes had promoter CpG sites that were both hypo‐ and hypermethylated, or mixed methylation (Figure 6B,C). Overall, the differences in myonuclear DNA methylation between young and old with short‐term MOV may relate to differences in the sustained transcriptional response (beyond 72 h) that ultimately leads to different magnitudes or rates of hypertrophy in aged muscle.

### 2.7 Methylome‐Transcriptome Integration Shows Epigenetic Control of the Muscle Transcriptome with Aging

Interpreting the impact of DNA methylation on gene expression can be complicated since many genes have regulatory regions with CpG sites that are of mixed methylation status (as shown in Figure 6). In an effort to decode this ambiguity, we used Binding and Expression Target Analysis (BETA), a multi‐omic integration tool that uses several genomic parameters that are relevant to inferring epigenetic control of transcription [84]. BETA was first adapted by us from ChIP‐seq analysis to understand how myonuclear DNA methylation regulates the transcriptome during acute MOV in young female mice [36]. In this prior work, BETA inferred the epigenetic regulation of metabolic‐related transcriptional adaptations in muscle during MOV, which we corroborated using high‐resolution respirometry [36]. We have since used this approach to understand how DNA methylation regulates the transcriptome throughout recovery from acute resistance exercise in humans [68], transcriptional remodeling after muscle injury when aged [85], as well as how the methylome may influence the proteome with late‐life exercise training in mice [86]. BETA generates regulatory scores for overall up‐ or down‐regulation and for individual genes, accounting for transcription start site (TSS) proximity and magnitude of differential methylation. In some cases, the BETA regulation score for up‐ or downregulation may not be statistically significant overall, but there can still be substantial regulation on a gene‐by‐gene basis, which we considered in our analysis.

By combining our RRBS and transcriptome datasets and focusing first on the effect of age (6–8 month vs. 24‐month sham mice), BETA inferred differential expression of 1,950 genes due to myonuclear DNA methylation (Figure S2A and File S3). Altered pathways of DNA methylation‐controlled genes largely reflected that of bulk RNA‐seq (Figure S2B,C), supporting the hypothesis that changes to the DNA methylome have appreciable regulatory influence on the muscle transcriptome as age progresses [81, 82, 83, 87, 81, 88].

### 2.8 Methylome‐Transcriptome Integration Suggests Greater Epigenetic Control of Muscle Growth‐Oriented Genes in Adult versus Aged MOV

**FIGURE 7:**
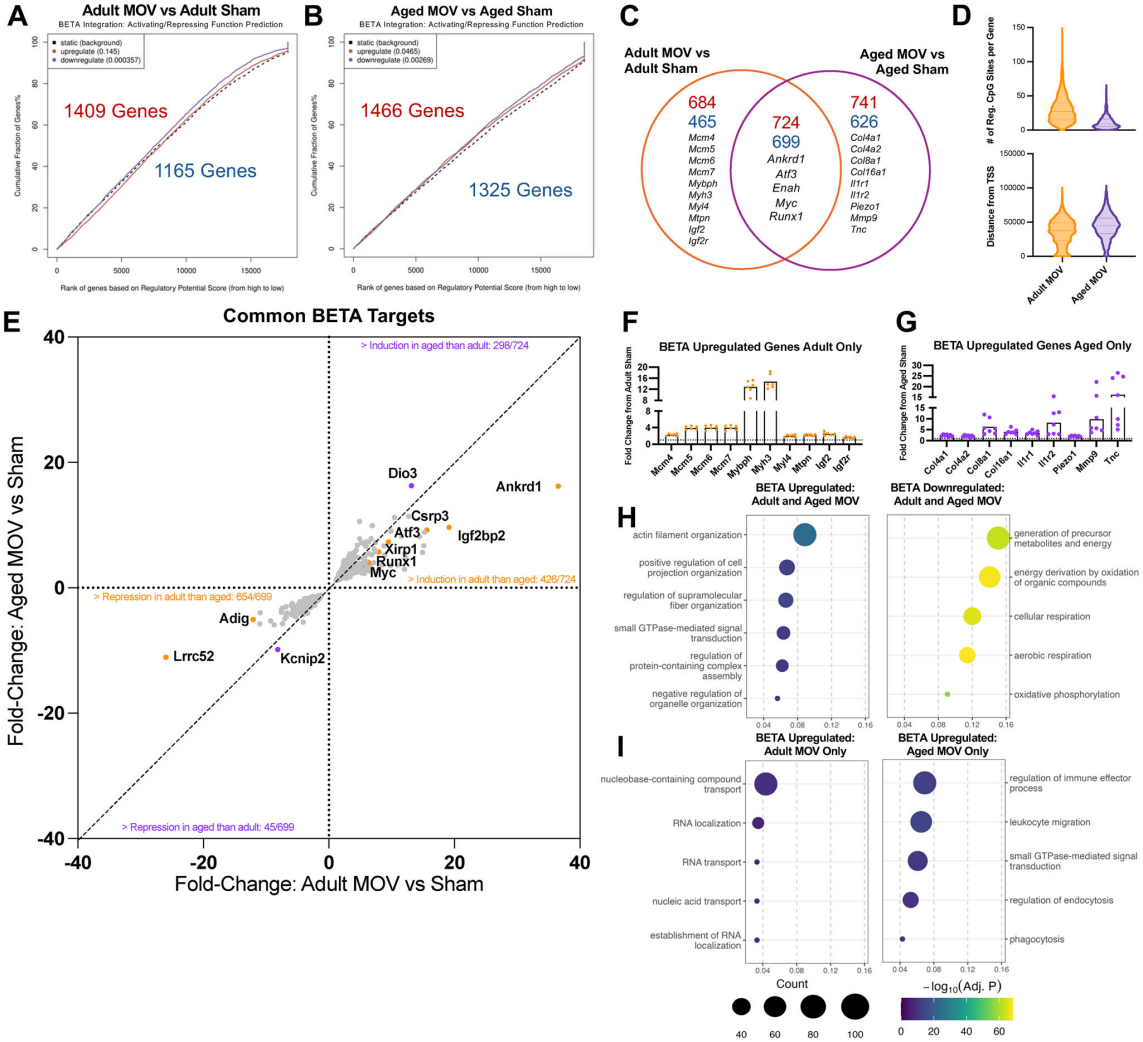
Omic integration of the myonuclear epigenome with muscle tissue RNA‐sequencing in adult and aged sham and MOV muscles. BETA integration analysis of myonuclear RRBS and bulk RNA‐seq data comparing up‐ and downregulated genes relative to background (significance indicated by *p*‐values in parentheses) for: (A) adult MOV vs adult sham, (B) aged MOV vs aged sham. (C) Venn diagram of genes regulated by methylation. (D) Violin plots showing # of regulatory CpG sites for each BETA target and average absolute distance from the transcription start site. (E) X‐Y plot showing fold changes of genes that are common BETA targets between adult MOV and aged MOV. (F) Plot of fold‐changes of select BETA targets exclusive to adult MOV, (G) Plot of fold‐changes of select BETA targets exclusive to aged MOV. (H) Top 5 enriched GO Biological Processes pathways from common upregulated and downregulated BETA targets. (I) Top 5 enriched GO Biological Processes pathways from upregulated BETA targets exclusive to adult MOV (left) and aged MOV (right). TSS = transcription start site. Reg = regulatory.

BETA predicted an overall similar number of genes to be regulated by DNA methylation in aged and adult mice in response to acute MOV (Figures 7A,B); however, the proportion of genes common to MOV in adult versus aged was only ∼36% (Figure 7C; File S3). Among common BETA target genes sharing the same overall pattern of expression (upregulated or downregulated in both ages), adult MOV featured more predicted regulatory CpG sites on average per gene, and the altered CpGs were relatively closer to the TSS compared to aged (Figure 7D). For example, in the BETA analysis, *Myc* in adult MOV had 10 predicted regulatory myonuclear CpG sites that were on average 826 base pairs (bp) from the TSS, but in aged MOV there were only 5 regulatory CpG sites that were on average 58,175 bp from the TSS (Figure S3A). Independent from BETA integration, myonuclear RRBS data alone corroborates age‐associated epigenetic effects on *Myc* after MOV with 11 significantly hypomethylated CpGs within the gene (promoter, intron, or exon) in adult whereas aged MOV had only a single hypomethylated CpG site (Figure S3B). This pattern holds for several other genes that had blunted transcriptomic responses to MOV when aged (see Figure 5G): *Myc, Runx1, Mybph*, and *Ankrd1* (Figure S3C). *Myc* expression is seemingly controlled by DNA methylation in other tissues and contexts [89, 90, 91, 92, 93], so differences in myonuclear DNA methylation around the *Myc* gene and others may explain blunted gene expression responses to MOV in aged versus adult. In general, a majority of common BETA targets exhibited greater fold‐changes in gene expression after MOV in adult versus aged (Figure 7E). GO pathway analyses of common BETA regulated genes in adult and aged points to upregulation of actin filament organization, signal transduction, and organelle organization (Figure 7H, left) with downregulation of mitochondrial aerobic respiration and oxidative phosphorylation (Figure 7H, right).

We next examined unique gene targets inferred to be regulated by DNA methylation during MOV in adult versus aged. There were 684 and 465 genes predicted to be up‐ or downregulated exclusively in adult MOV, respectively (Figure 7C). Adult MOV featured predicted methylation regulation of members of the minichromosome maintenance family: *Mcm4, Mcm5, Mcm6, Mcm7*. These genes are implicated in initiation of DNA replication in eukaryotes. Minichromosome gene regulation in adult myonuclei but not aged is provocative since it could be related to *de novo* resident myonuclear DNA synthesis observed during MOV‐induced muscle growth in young mice [94, 95, 96]. If the ability to synthesize myonuclear DNA with MOV is compromised with age, perhaps it is due to differential regulation (and perhaps altered long‐term induction) of *Mcm* genes. Myosin binding protein H (*Mybph*) was upregulated and predicted to be controlled by methylation only in adult MOV (Figure 7F). *Mybph* is upregulated in peripheral artery disease (PAD) and the neurodegenerative disease amyotrophic lateral sclerosis (ALS) [97, 98], possibly as a compensatory response to atrophy and dysfunction. At the pathway level, BETA predicted genes exclusive to adult MOV corresponded to RNA and nucleic acid transport (Figure 7I, left). There were 741 up‐ and 626 downregulated genes predicted to be controlled by methylation exclusive to aged MOV (Figure 7C). These genes were related to ECM and inflammation/immune process pathways, with leukocyte migration and phagocytosis being the top enriched gene ontologies (Figure 7I, right). Specifically, aged muscle was enriched for the collagen genes *Col4a1, Col4a2, Col8a1*, and *Col16a1* and interleukin receptors *Il1r1* and *Il1r2* with MOV (Figure 7G). We previously reported robust induction of collagen and ECM remodeling genes in myonuclei during MOV in young mice (∼2‐month‐old), but the age‐specific methylome‐transcriptome signature observed here may relate to impaired ECM adaptation during loading when aged [49].

### 2.9 Single Myonucleus RNA‐Sequencing (smnRNA‐seq) in Adult versus Aged Muscle Reveals Age‐Dependent Regulation of Innervation and *Nr4a3* Genes, Which Are Linked to Myonuclear DNA Methylation

**FIGURE 8:**
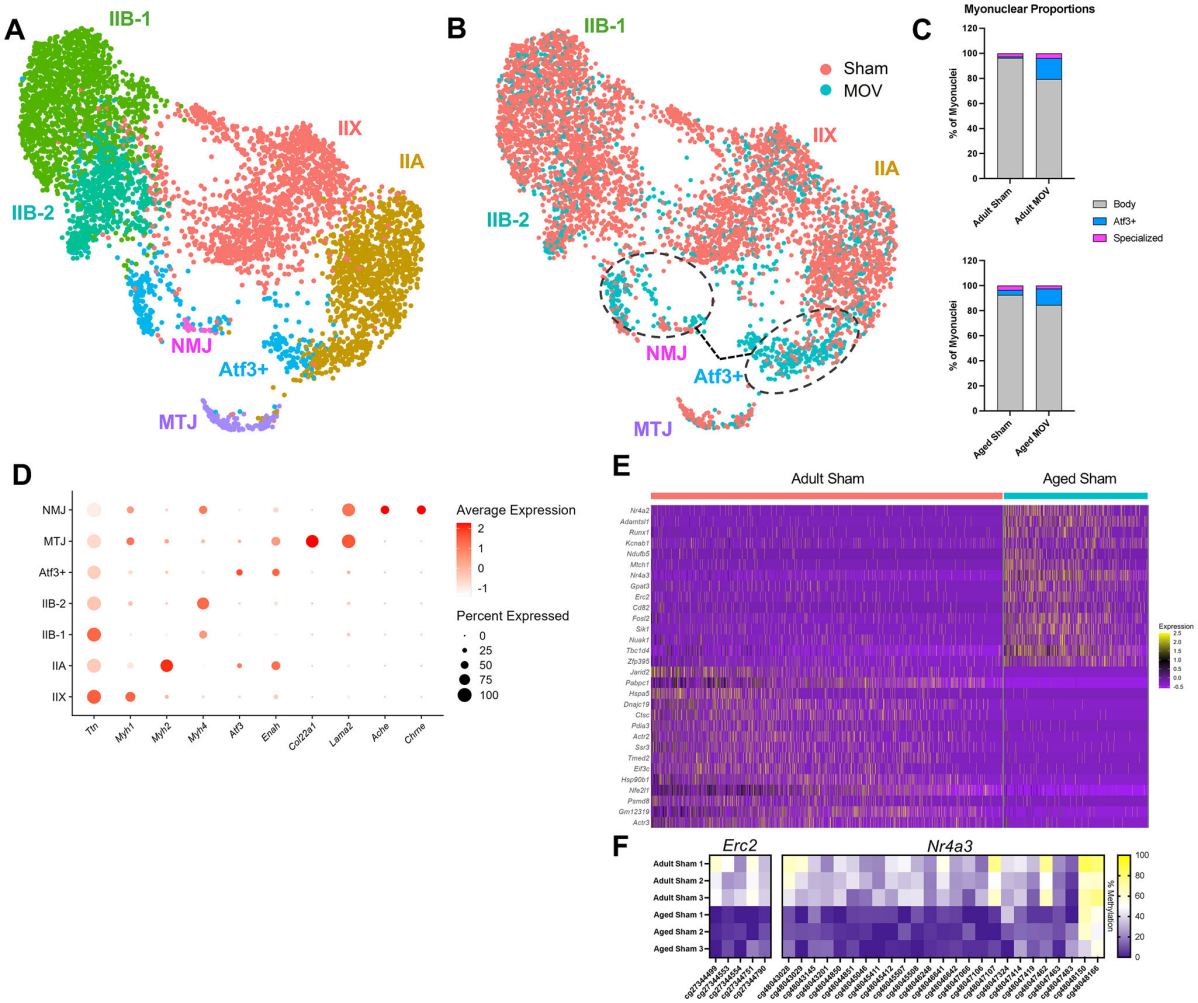
Single myonucleus RNA‐sequencing (smnRNA‐seq) in adult and aged sham and MOV muscles with a focus on the effect of aging using the sham conditions. (A) Integrated UMAP of integrated all experimental conditions, labeled/colored by cluster. (B) Split UMAP of each experimental condition colored by cluster. (C) Percent composition of adult and aged myonuclei by cluster (D) Dot plot depicting top differential marker genes for each myonuclear cluster. Dot size represents the percentage of nuclei expressing the gene. (E) Heatmap of top 20 up‐ and down‐regulated genes between aged sham and adult sham. (F) Heatmap showing myonuclear DNA methylation percentage between aged sham and adult sham for select upregulated genes. NMJ = Neuromuscular Junction. MTJ = Myotendinous Junction. Body = Combined myosin expressing clusters. Differentially expressed genes between clusters and/or experimental conditions were identified using the FindAllMarkers() or FindMarkers() functions, which uses a Wilcoxon Rank Sum Test, with min.pct = 0.25, Log_2_FC > 0.25, with adj. *p* (FDR) < 0.05.

We next profiled the transcriptomes of 5,436 myonuclei using smnRNA‐seq (Figure 8A, 716‐2,797 per experimental condition, File S4). Initially, data from all experimental conditions were integrated and used to derive myonuclear clusters (Figure 8A). UMAPs grouped by MOV versus sham, irrespective of age, are shown in Figure 8B. Four clusters were defined by myosin heavy chain gene expression (*Myh2, Myh1, Myh4*). These clusters were grouped together, termed “body myonuclei”, and comprised ∼90%–95% of myonuclei in sham conditions (Figure 8C). Two of the seven myonuclear clusters were from specialized compartments such as the myotendinous junction (MTJ, ∼2.5%) and neuromuscular junction (NMJ, ∼0.6%). MTJ myonuclei were characterized by high expression of *Col22a1, Lama2, App*, and *Fras1*, and NMJ myonuclei by genes such as *Ache, Chrne, Ano4, Etv5*, *Vav3*, and *Col4a3* (Figure 8D). The remaining cluster was an “*Atf3*+” population which expanded by ∼15% in MOV groups compared to sham (Figure 8D; Figure S4 and File S4).

We first defined the difference in aged sham versus adult sham myonuclei at single nucleus resolution to assess the effect of aging. We found enrichment of *Erc2, Nr4a3*, and *Runx1* in aged myonuclei, among other genes (Figure 8E). According to BETA, *Erc2* upregulation was associated exclusively with hypomethylated regulatory CpGs located in close proximity to the TSS in myonuclei (<360 bp, Figure 8F). Elevated *Erc2* was recently implicated in reduced muscle mass with aging and plays a role in organizing presynaptic active zones [98, 99, 100]. The *Nr4a* nuclear receptor family genes are among the most highly exercise responsive genes in human muscle, serving as a critical regulator of glucose and lipid metabolism [101, 102, 103]. Downregulation of *Nr4a* genes is deleterious for muscle mTORC1 signaling, ribosome biogenesis, and protein synthesis in muscle [101, 102, 104, 105]. Upregulation of *Nr4a3* is typically associated with exercise adaptation and is downregulated with inactivity in human muscle [101]. The link with aging is less well‐known. Our bulk RNA‐seq data do not show this family of genes to be altered with age; however, previous reports using single nucleus RNA‐seq (snRNA‐seq) and fluorescent in situ hybridization (snRNA‐FISH) show *Nr4a3* to be upregulated in aged tibialis anterior and gastrocnemius muscle [106]. Accordingly, *Nr4a3* was robustly hypomethylated in aged myonuclei relative to adult in our data (Figure 8F). Upregulation of *Nr4a3* with age could be specific to the ultra‐fast glycolytic myosin types found in mice given *Nr4a3* is downregulated in aged human muscle, which is generally comprised of mixed slow and fast‐oxidative myosin types [107]. Induction of *Runx1* is associated with muscle denervation during cachexia and aging in muscle [107, 108, 109]. Myonuclear *Runx1* induction aligns with observations of the appearance of denervated fibers with aging, specifically in fast‐glycolytic subtypes [110, 111]. *Runx1* was not predicted to be regulated by BETA with aging alone, albeit there was considerable differential methylation (both hypo‐ and hypomethylation) at promoter and intron regions (Figure S3).

### 2.10 Age‐Dependent Myonucleus‐Specific Gene Expression Revealed by Acute MOV in Adult and Aged Muscle

Only one previous investigation has performed single myonucleus RNA‐sequencing (smnRNA‐seq) with MOV, and this was in young mice [112]. There are several notable differences in experimental design to consider between our study and the previous study. First, in the previously published study, the myonuclear GFP labeling period was prior to and during MOV. This strategy captures newly fused satellite cells in addition to resident myonuclei. In the current study, resident myonuclei were labeled prior to MOV, thus excluding satellite cell‐derived myonuclei during FANS isolation. This difference in experimental design is important given the prior study used 3‐month‐old mice, an age at which mice may still be undergoing developmental muscle growth and be reliant on satellite cell fusion and myonuclear accretion for initiating muscle hypertrophy compared to mice >4 months of age [18, 113, 114]. Our experiment was conducted in fully mature adult mice (>6 months). Second, in the prior study, the MOV period was 7 days versus our 3 days. We are evaluating the inherent ability of resident myonuclei to support hypertrophy largely independent from significant satellite cell fusion, which tends to occur later during MOV [33] but can happen to some degree by three days due to direct fusion to muscle fibers without a round of proliferation [115, 116]. It is also worth mentioning that our smnRNA‐seq was carried out on portions of the same muscles used for bulk RNA‐seq and myonuclear RRBS, which facilitates our ability to validate conclusions across assays.

**FIGURE 9:**
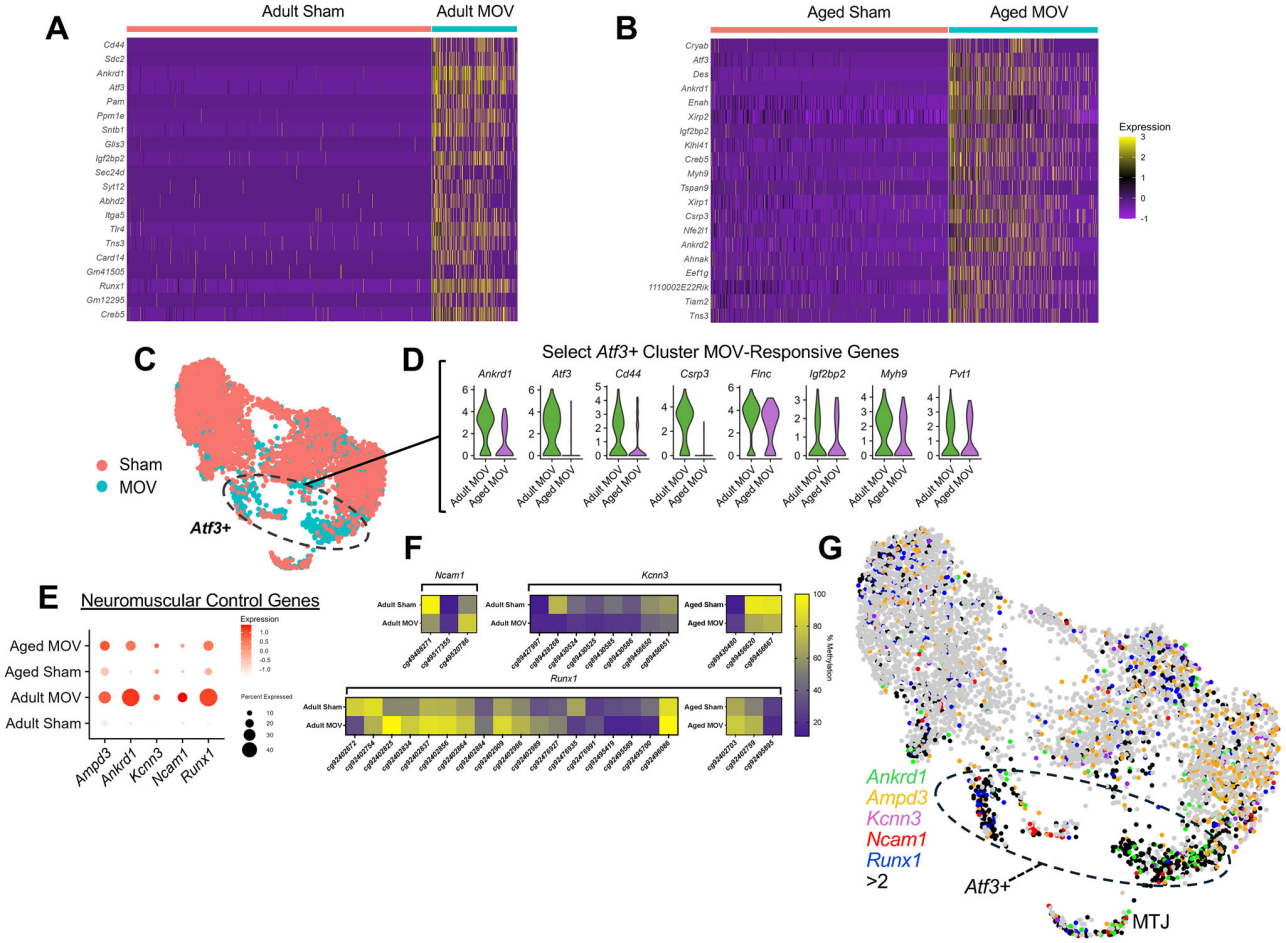
The adult versus aged transcriptome response to MOV at single myonucleus resolution and comparison to myonuclear DNA methylation data. (A) Heatmap of top 20 upregulated genes between adult MOV and adult sham. (B) Heatmap of top 20 upregulated genes between aged MOV and aged sham. (C) UMAP grouped by MOV vs Sham. (D) Violin Plot showing expression of select genes upregulated in the Atf3+ population during MOV. In adult MOV, most genes are significant adj. *p* < 0.05. In aged MOV, no genes reached adj. *p* < 0.05 or were ns at *p* < 0.05. (E) Bubble plot showing expression of select genes involved in neuromuscular junction control. (F) Heatmap showing myonuclear DNA methylation percentage between MOV and sham conditions for neuromuscular control genes. (G) UMAP feature plot of neuromuscular control genes. Nuclei expressing >2 of the listed genes are colored black. ns = not significant. Differentially expressed genes between clusters and/or experimental conditions were identified using the FindAllMarkers() or FindMarkers() functions, which uses a Wilcoxon Rank Sum Test, with min.pct = 0.25, Log_2_FC > 0.25, with adj. *p* (FDR) < 0.05.

With the modest number of myonuclei in our analysis, we first chose to broadly define how adult and aged myonuclei respond to MOV independent of cluster. Many of the top upregulated genes with MOV were common to adult and aged myonuclei: *Atf3, Ankrd1, Igf2bp2*, and *Creb5* (Figures 9A,B). Still, many genes were uniquely upregulated in adult versus aged MOV such as *Cd44, Pam*, and *Runx1*. *Cd44* is necessary for proper muscle regeneration. *Cd44* knockout results in reduced muscle size and delayed recovery after muscle injury [117]. Perhaps higher *Cd44* in adult versus aged myonuclei with MOV contributes to age‐specific muscle plasticity in a yet undefined way.

We next focused on the *Atf3*+ population because it expands with MOV from ∼2% of myonuclei in sham to ∼15% of myonuclei in MOV in both ages (Figures 8C and 9C; File S4). Cluster‐level analysis on this population showed *Ankrd1, Atf3, Cd44, Csrp3, Flnc, Igf2bp2, Myh9*, and *Pvt1* were relatively more induced in adult versus aged MOV (Log_2_Fold changes relative to respective sham; *Ankrd1*: 9.9 vs 4.9, *Atf3*: 10.1 vs 8.6; *Cd44*: 8.3 vs 4.0; *Csrp3*: 6.3 vs 2.0; *Flnc*: 3.8 vs 2.2; *Igf2bp2*: 5.7 vs 4; *Myh9*: 3.0 vs 3.1, *Pvt1*: 1.3 vs 0.9 in adult versus aged, respectively) (Figure 9D). Enrichment for several of these genes is similar to what is found in an *Atf3+* “sarcomere assembly” population defined previously during skeletal muscle development [106] and in response to MOV in young mice [112]. Some of these same genes are enriched in resident myonuclei that migrate to sites of focal muscle fiber membrane damage [118]. Perhaps this emergent resident population with MOV are migrating “injury repair” myonuclei [118, 119], and higher expression of these genes in adults contributes to superior sarcolemma repair versus aged. The lncRNA *Pvt1*, which was upregulated during MOV in adult myonuclei but not in aged myonuclei, can interact with and stabilize *c‐* and *n‐Myc* (Figure 9D) [120]. These specific myonuclei may be part of an early myofibrillogenesis program that occurs during rapid developmental muscle growth (circa post‐natal day 21) as well as MOV observed here, with a stronger response in adult versus aged. The induction of *Igf2bp2* (Insulin‐like growth factor mRNA binding protein 2) in the *Atf3*+ population is intriguing. *Igf2bp2* binds *Igf2*, and *Igf2* was previously shown to be enriched in a myonuclear population derived from fused muscle satellite cells during MOV [112]. Although this *Igf2+* population was not detected in our dataset due to the aforementioned methodological differences versus the prior work, complementary expression of these two genes may reflect a synergy between two distinct myonuclear populations (resident and satellite‐cell derived) to coordinate the response to MOV.

Also in *Atf3*‐high myonuclei, we found upregulation of genes previously implicated in muscle innervation processes (*Ampd3, Ankrd1, Kcnn3, Ncam1, Runx1*, Figure 9E), and to a relatively greater extent in adult versus aged MOV. All of these genes were predicted to be regulated by methylation in myonuclei, with *Kcnn3, Ncam1, and Runx1* associated with myonuclear hypomethylation of promoter regions (Figure 9F). *Ampd3* and *Ankrd1* have methylation signatures at sites more distant from the TSS (see File S3). When overlaid on the UMAP, these genes show high co‐expression (black dots, >2 genes expressed in each nucleus) across the MOV responsive *Atf3*+ population, as well as MTJ myonuclei (Figure 9G) [106, 108, 109]. We did not identify myonuclei enriched for *H19*, *Igf2*, *Myh3* and *Myh8* (embryonic and neonatal myosin heavy chains) as observed previously during a longer duration of MOV in young mice [112]. The absence of these nuclei emphasizes that the emergence of these genes is a feature of recently fused satellite cells, which were deliberately excluded from our dataset (File S4).

## 3 Discussion

Aged muscle generally has a reduced capacity to grow in response to a hypertrophic stimulus [7, 8, 9, 10, 11]. Understanding the mechanisms underlying an age‐associated loss of muscle plasticity is complicated by the syncytial nature of myofibers and the influence of non‐muscle cell types that can obscure myonuclear‐specific changes. Parsing the roles of resident versus satellite cell‐derived myonuclei is also important since resident myonuclei initiate the muscle hypertrophic process [14]. Taking advantage of the genetically modified doxycycline‐inducible HSA‐GFP mouse model [12, 15], which enables the isolation of a high‐purity population of resident myonuclei, in addition to myonuclear DNA methylation analysis and bulk RNA‐sequencing, we evaluated the myonuclear molecular responses during the early phase of loading‐induced muscle growth across several molecular layers. These datasets will serve as a resource for those at the intersection of skeletal muscle biology and aging research.

In sham mice, multi‐omic integration revealed aged myonuclei experience broad changes in the transcriptome that is likely controlled by DNA methylation. Aging was associated with higher expression of ribosomal and mitochondrial gene expression and lower expression of ECM–related genes concomitant with changes to the DNA methylome in these same genes. Elevated ribosome gene regulation with age could be related to higher protein synthesis and dysregulated proteostasis that occurs during the muscle aging process [27]. A possible accumulation of dysfunctional ribosomes could also affect ribophagy, the autophagic degradation process for ribosomes, and compromise translational capacity in response to an anabolic stimulus [121]. Aged sham myonuclei have elevated expression of the transcription factor *Runx1* relative to young adult*. Runx1* is upregulated in denervated muscle [107, 108]. Higher *Runx1* with age supports previous observations in aging humans suggesting an increased number of denervated fibers, specifically fast muscle fibers [122, 123, 124, 125]. Altered innervation could contribute to a compromised ability to respond to a muscle growth stimulus. In previous work, an *Ampd3+* population of myonuclei appears in 30‐month‐old mice, which was posited to be a dysfunctional denervated state given they were co‐expressing pro‐atrophy and proteasome‐related genes [106]. Our data broadly align with a molecular signature of denervation from aging that is discernable at the epigenetic level in myonuclei of mice.

By integrating myonuclear methylome and bulk transcriptome data, we provide the first detailed information on the myonuclear molecular landscape following an acute hypertrophic growth stimulus in adult versus aged mice. A similar number of genes had coordinated epigenome‐transcriptome regulation after MOV in adult and aged muscle (∼1,400 genes, 36% overlap). Our bulk RNA‐sequencing show adult and aged muscle have a large conserved set of genes responsive to MOV at this early timepoint, including genes involved in cytoskeletal organization and immune signaling along with suppression of oxidative phosphorylation pathways, as we have previously shown [36, 49]. *miR‐1* emerged as a key regulator of this metabolic reprogramming, being strongly repressed by MOV in both ages. With our inducible knockout of *miR‐1* in adult muscle, we provide evidence that this myomiR is a powerful regulator of the skeletal muscle transcriptome. Its repression explains some of the commonalities in the transcriptomes of adult and aged MOV, specifically as it relates to metabolism gene expression.

Although there were transcriptional profiles common to MOV‐induced gene expression among adult and aged at the tissue level, aged muscle tended to have a blunted response. Our data suggest this could be attributed to a few factors: 1) the change in percent methylation of DM CpG sites in aged MOV myonuclei were generally much lower than adult, 2) there are on average fewer regulatory CpG sites per target gene in aged, and 3) regulatory CpG sites tend to be further from the transcription start site (or promoter region) in aged. Combined, these factors may lead to aged myonuclei having less control over each gene, potentially constraining the transcription required to sustain and maximally adapt to an anabolic stimulus. To this point, aged muscle displayed a weaker induction of myonuclear enriched genes previously implicated in muscle mass regulation, including *Ankrd1*, *Mybph*, *Myc*, and *Runx1*. Instead, aged muscle showed a stronger induction of inflammatory, immune system, and cytokine‐oriented genes with MOV. There are also numerous genes implicated in muscle mass regulation that were uniquely regulated at the methylation level in adult versus aged MOV muscle—such as minichromosome maintenance genes—that may contribute to eventual differential adaptation between adult and aged.

Our smnRNA‐seq data reinforce previous work showing expansion of an *Atf3*‐enriched myonuclear subpopulation after overload [112]. This pattern was observed in both age groups; however, some genes enriched in this cluster generally exhibited weaker induction in aged than adult. The *Atf3*‐enriched population may be an early myofibrillogenesis or sarcomere assembly program that is similar to what emerges in post‐natal muscle growth [106]. Adult muscle also featured stronger induction and epigenetic coordination of genes associated with neuromuscular remodeling: *Ampd3, Ankrd1, Kcnn3, Ncam1*, and *Runx1*. These observations, in context with our prior bulk myonuclear RNA‐seq in young mice [49, 106] and the observation that some of these genes are also upregulated by aging alone, raises a few possibilities: 1) acute MOV causes rapid neuromuscular junction (NMJ) remodeling that induces a de/re‐innervation‐like signature; 2) these “neuromuscular” genes are highly pleiotropic and act in a condition‐dependent context to regulate muscle remodeling independent from NMJ disruption, or 3) all events are happening simultaneously. In the case of *Runx1* for instance, its induction can be sufficient for myotube hypertrophy via an mTORC1‐associated protein synthesis mechanism [126] which may or may not be related to its role in innervation. Nevertheless, the stronger induction of NMJ‐related genes in adult versus aged MOV suggests an age‐specific myonucleus‐controlled neural contribution to the early hypertrophic process, which is further supported by our cellular deconvolution data.

There are a few limitations to our study that are worth considering. Our study only used male mice. We cannot confirm whether these results would be replicated in female mice. Additionally, we are capturing the early stage of the muscle response to MOV, where myofibrillar growth has not yet ensued. This time point may not necessarily reflect later molecular responses. Analyzing later time points and using different resistance exercise models may be helpful for validating the molecular signatures we observe, as well as age‐related differences in methylation/gene expression. Lastly, our smnRNA‐seq is a limited dataset that did not allow us to fully evaluate the myonuclear transcriptome at high resolution and coverage; however, usage of the same muscles for all analyses increases the robustness of our conclusions, as does agreement with previously published smnRNA‐seq and bulk myonuclear RNA‐seq datasets from young animals [49, 112]. Limitations aside, our integrated multimodal datasets provide unprecedented and detailed information on resident myonucleus‐specific hypertrophic responses and could lead to new therapeutic targets for enhancing muscle adaptability in old age.

### 3.1 Translational Perspectives

Our preclinical studies suggest that MOV‐induced skeletal muscle hypertrophy is impaired in mice ≥22 months old [17, 127]. We are unsure if this diminished plasticity occurs earlier than 22–24 months (e.g. in later life at 18 months of age), or if the MOV hypertrophic process is merely delayed versus inexorably blunted with age. We do not report on morphological differences to MOV according to age in the current investigation, although we have shown these data previously using later time points [127]. Exercise training volume and intensity is usually diminished with aging which may in part explain hypertrophic unresponsiveness as age progresses [10]; however, even when efforts are made to equate loading between young and aged, muscle adaptation is still lower in aged [71, 128, 129, 130]. Humans aged 85–97 years can retain a capacity for resistance training‐induced hypertrophic potential [131], but this is not universally reported in the literature [7, 8, 132, 133]. Discrepancies in adaptive potential to resistance training in aged human skeletal muscle reported in the literature could be related to a variety of factors including lifestyle, diet, geographic region, training duration, and/or the training stimulus, among other factors. There is heterogeneity in training responsiveness of both younger and older humans which is complex to unravel [133, 134, 135, 136, 137, 138]. Perhaps this heterogeneity in aged humans is related to age‐associated dysregulation of the immune system which conditions the muscle environment and can influence aspects of muscle plasticity [139, 140, 141]. Regardless, our findings suggest that impaired skeletal muscle adaptability that is known to occur in an animal model of mechanical loading is linked to age‐associated changes and responses specifically within myonuclei.

## 4 Methods

### 4.1 Animals

All animal procedures were approved by the University of Arkansas IACUC. Mice were housed in a temperature and humidity‐controlled room, maintained on a 12:12‐h light‐dark cycle, and food and water were provided ad libitum throughout experimentation. At euthanasia (morning, ZT 1–5), animals were first deeply anesthetized with isoflurane and sacrificed via cervical dislocation. Adult (6–8 months of age) and aged (24 months of age) male human skeletal actin reverse tetracycline transactivator—tetracycline response element histone 2B green fluorescent protein (HSA‐rtTA^+/−^;TRE‐H2B‐GFP^+/−^, or HSA‐GFP) mice were generated and genotyped as previously described by us [12, 15]. HSA‐GFP mice were treated with low‐dose doxycycline (0.5 mg/ml doxycycline in drinking water with 2% sucrose) for 7 days to induce GFP labeling of myonuclei, followed by a washout period (normal drinking water) of at least 7 days. This strategy leads to the labeling of ∼95% of myonuclei with minimal off‐target labeling of non‐myonuclei. To knockout *miR‐1* in adult mouse skeletal muscle fibers, skeletal muscle‐specific inducible *Mer‐Cre‐Mer* (HSA‐MCM) mice were crossed with *miR‐1‐1^fl/fl^; miR‐1‐2^fl/fl^* (*miR‐1^fl/fl^*) mice to produce HSA‐MCM^+/−^; *miR‐1‐1^fl/fl^; miR‐1‐2^fl/fl^* mice (termed HSA‐miR‐1, KO) [40, 135]. HSA‐MCM^−/−^ *; miR‐1^fl/fl^* littermates mice served as controls (CON).

### 4.2 Synergist Ablation Mechanical Overload Experiment

Synergist ablation mechanical overload (MOV) of the plantaris was performed as previously described by our lab group at a consistent daily interval (ZT 1–5) [12, 49]. Briefly, synergist ablation is a surgical procedure that occurs while mice are under anesthesia. The surgery involves making an incision on the posterior aspect of the lower hindlimb, cutting of the Achilles tendon followed by careful removal of ∼30% the gastrocnemius–soleus complex while leaving the plantaris muscle and tendon intact. Sham surgery (control) involved all the steps of synergist ablation but no tendon is cut or muscle is removed. Following surgery mice resumed ambulatory cage activity. Euthanasia was performed 72 hours after MOV and both plantaris muscles were dissected and immediately flash frozen in liquid nitrogen. All muscle was used for downstream molecular analyses across four experimental conditions: adult sham (*n =* 6), adult MOV (*n =* 6), aged sham (*n =* 7), aged MOV (*n =* 7). The same cohort of animals were used to perform all experiments reported (Figure 1).

### 4.3 Inducible Muscle‐Specific miR‐1 Knockout Experiment

Middle‐aged (10–12‐month‐old) male HSA‐miR‐1 and *miR‐1^fl/fl^* mice (*n* = 3/group) were administered tamoxifen (2 mg/day) by intraperitoneal injection for five consecutive days, followed by an 8‐week chase period. At euthanasia, KO and CON mice had all lower hindlimb muscle rapidly dissected, with one limb flash frozen for molecular analyses. Plantaris muscle was used for analyses.

### 4.4 RNA Isolation, cDNA Synthesis, and Gene Expression Analysis

RNA was isolated from approximately one‐third of each plantaris muscle (∼10 mg) using TRIzol Reagent (Sigma‐Aldrich, St. Louis, MO, USA). Tissue was homogenized using zirconia beads and the Fisher Bead Mill (Fisher, Hampton, NH, USA). Following homogenization, RNA was isolated via phase separation by addition of chloroform and then centrifugation. The aqueous phase was transferred to a new sterile tube and further processed on spin columns according to manufacturer instructions using the Direct‐zol Kit (Zymo Research, Irvine, CA, USA). RNA quality was checked on Agilent TapeStation with RNA screentape to confirm quality and purity. RNA integrity number (RIN) was >7 for all samples (8.3±0.7). For RT‐qPCR of *miR‐1* expression, cDNA was synthesized using the TaqMan MicroRNA Kit (4366596, Thermo Fisher Scientific, Waltham, MA). Gene expression of *miR‐1* was analyzed by qPCR using TaqMan MicroRNA Assays (4427975, Thermo Fisher) as follows: *miR‐1* (Assay ID 002222) and *U6* snRNA as the endogenous control for normalization (Assay ID 001973). The 2ˆ‐(∆∆Ct) method was used to calculate fold change.

### 4.5 RNA Sequencing, Data Processing, and Visualization

RNA was sequenced by Novogene on an Illumina HiSeq using 150 bp paired‐end sequencing, as we have previously done [49]. Raw FASTQ files were processed in Partek Flow as described below in the Statistical Analyses section. Pathway analyses were performed on up‐ and downregulated DEGs in R Studio (version 2025.09.0.387) with the 2025 gene ontology (GO) database as our cross reference (GO.db: Bioconductor version 3.21). We used all protein‐coding genes detected in our RNA‐sequencing dataset as our background correction for the pathway analysis [142]. For comparison of adult and old MOV RNA‐seq data to myonuclear RNA‐seq data during MOV, the list of upregulated genes from a previous study was used as a reference [48]. Figures were generated in GraphPad Prism version 10.6 for Mac OS X (GraphPad Software, La Jolla, CA), RStudio, and BioRender.

### 4.6 Digital Deconvolution of Cell Composition Using Granulator

Cell type abundance was predicted from bulk RNA‐sequencing data using Bioconductor R package Granulator (https://bioconductor.org/packages/release/bioc/html/granulator.html) [50]. Referencing single cell RNA‐sequencing data, Granulator infers cell type abundance by modeling gene expression levels as weighted sums of the cell‐type specific expression profiles. We used skeletal muscle single‐cell RNA‐seq data from 4‐day murine plantaris tenotomy data from Zhang et al. [51] (see File S5). The publicly available datasets (10X Genomics.h5 files) were downloaded from GEO (GSE232257), reanalyzed with Seurat, and cell clusters were identified using the exact parameters outlined in the initial publication. Normalized gene expression matrices of each cell type serving as a reference gene expression matrix was integrated with normalized counts from our bulk RNA‐sequencing data, and cell proportions were predicted by Granulator.

### 4.7 Fluorescent Activated Nuclear Sorting (FANS)

Myonuclei were isolated via fluorescent activated nuclear sorting (FANS) on a MACSQuant Tyto Cell Sorter (Miltenyi Biotec, Bergisch Gladbach, Germany). For myonuclear DNA methylation experiments, approximately one‐half of each plantaris was used (∼15 mg). Muscle was placed in a small glass beaker with a sucrose‐based buffer mimicking physiological conditions (5 mM PIPES, 85 mM KCl, 1 mM CaCl_2_, 5% sucrose, 2X HALT Protease inhibitors, and 0.25% NP‐40), pulsed with 4 µL of propidium iodide, then minced with scissors until a slurry. The nuclear suspension was then transferred to glass Dounce for further manual homogenization, then strained through a 20‐µm MACSQuant pre‐separation filter directly into MACSQuant Tyto regular‐speed sorting cartridge (Cat #: 130‐104‐791). For single myonucleus RNA‐sequencing experiments, remaining muscle (∼2–3 mg) of plantaris from three mice per condition were pooled in homogenization buffer (500 µL HEPES [1 m], 3 mL KCl [1 m], 250 µL spermidine [100 mM], 750 µL spermine tetrahydrochloride [10 mM], 10 mL EDTA [10 mM], 250 µL EGTA [100 mM], 2.5 mL MgCl [100 mM], 5.13 g sucrose) with 0.2U/µL RNAse inhibitors (Protector RNase Inhibitor, Millipore Sigma, Burlington, MA, USA). The muscle was minced in buffer with scissors on ice in a low‐bind 1.5 mL tube, dounced ∼20 times with a plastic pestle with a gentle twist at the bottom, then strained through a 20‐µm MACSQuant pre‐separation filter (Cat #: 130‐101‐812) directly into MACSQuant Tyto high‐speed sorting cartridge (Cat #: 130‐121‐549). In both experiments, FANS gating was established to exclude debris and identify nuclei positive for both GFP (intrinsic myonuclear label) and propidium iodide for DNA (PI) then sorted directly into the respective buffer for downstream analyses. Myonuclei for DNA methylation analyses were sorted into ATL buffer and proteinase K (Qiagen) for genomic DNA purification. Myonuclei for smnRNA‐sequencing analyses were sorted into PBS/1% BSA/RNase inhibitors.

### 4.8 Myonuclear Genomic DNA Isolation, Reduced Representation Bisulfite Sequencing, and Analysis

DNA isolation was carried out according to a previous protocol described by us with minor adjustments [10]. Briefly, using the QIAamp DNA micro kit (Qiagen, Hilden, Germany), myonuclei sorted into buffer ATL and proteinase K were incubated for a minimum of 4 h at 56°C. DNA binding to the column was conducted using 1 µg of carrier RNA, and washes and centrifugations were carried out according to the manufacturer's instructions. DNA was eluted in 12 µl of nuclease‐free H_2_O, quality checked on the Agilent Tapestation with the genomic DNA (gDNA) screen tape and placed in −20°C until later analyses. Low‐input Msp1 Reduced Representation Bisulfite Sequencing (RRBS) was performed by Zymo Research using 5 ng of gDNA that was generally >40,000 base pairs (bp) in length. Some samples did not reach the minimum gDNA mass requirement for RRBS and could not be included in downstream analysis. Quality control and adapter sequence trimming were performed using FastQC and Cutadapt, respectively as parts of the Trim Galore wrapper. Low‐quality base calls (Phred score <20) were removed prior to trimming adapter sequences. Bismark aligner was used to align the sequence reads to the bisulfite‐converted mm39 genome prior to data processing. Coverage (.cov) ds produced from Bismark aligner were used for data analysis in the methylKit R package, with a minimum reads cut off of >10x coverage per CpG site across all samples and a minimum base coverage of 1 per sample, as previously described [12, 68, 143]. Percent methylation and percent differential methylation were then obtained from methylKit following analysis.

### 4.9 BETA Integration Pathway Analysis

Integration of differential DNA methylation and gene expression data was performed using BETA basic (Binding and Expression Target Analysis, v1.0.7). This software provides an integrated analysis of transcription‐factor binding to genomic DNA and transcript abundance using chromatin immunoprecipitation sequencing (ChIP‐seq) and transcriptomics (RNA‐seq) datasets [84]. BETA models the effect of regulatory elements using a natural‐log function of their distance to the transcription start site (TSS) to calculate a regulatory potential score [144]. This method for integration of RRBS and RNA‐seq data using BETA is consistent with our previous publications [36, 68]. In this study, differentially methylated CpG sites derived from RRBS were formatted as BED “peak” files and used as the regulatory‐element input, while differentially expressed genes (adjusted p < 0.05) from bulk RNA‐seq were used as the expression input. Gene annotations were derived from the mm39 UCSC GTF converted to BED format. The BETA basic was executed with the following command: “‐k BSF –gname2 –df 0.05 –pn 100000 ‐c 0.05”. These parameters specify the binding‐site kernel function, use of gene symbols, a 5% FDR cutoff for both methylation and expression data, and 100,000 permutations for robust significance estimation. CpG‐associated peaks within 100 kb of a gene's TSS were included in the calculation of the regulatory potential through the following equation:

$$\;s_{g}=\sum_{i=1}^{k}e^{\left\{-\left(0.5+4\Delta_{i}\right)\right\}}$$

In this formulation, *k* represents the number of CpG‐associated peaks (CpG islands) linked to gene *g*, and *Δ_i_* represents the distance of each element from the TSS, inversely scaled so that elements nearer the TSS exert greater influence on the regulatory potential score (*s_g_*). Genes were classified as activated, repressed, or non‐targeted according to the direction of their expression change relative to the predicted regulatory potential. It is also worth mentioning not all CpG‐associated peaks predicted to regulate gene activation or repression are found to be differentially methylated, and vice versa.

### 4.10 Single Myonucleus RNA Sequencing Library Preparation and Analysis

For this analysis, plantaris muscle from 3 samples were pooled per condition for analysis – MOV versus sham in adult and aged (*n =* 4 samples). GFP/PI+ myonuclei were isolated via FANS on our MACSQuant Tyto Sorter and sorted directly into 36 µl of PBS/1% BSA/RNase inhibitors to minimize dilution. This was the final analysis performed from the same cohort of mice, so at this stage we were limited to ∼2–3 mg of plantaris muscle per mouse. Combined with more gentle tissue dissociation methods compared to isolation of myonuclei for genomic DNA isolation (plastic pestle versus glass Dounce) in an effort to preserve nuclei integrity, the resulting nuclei yield per unit mass of tissue mass was lower than the maximum input into the 10X Chromium device. We combined 36.6 µl of the myonuclei suspension with 28.4 µl of reverse transcription master mix (MM). The 65 µl myonuclei + MM solution was loaded into the GEM‐X 3’ chip for GEM formation, then the library was prepared using the Single Cell 3’ Reagent Kit v4 according to the manufacturer's protocol. Following library construction, libraries were sequenced on the Illumina Nova NextSeq X Plus System by Novogene to 200 million reads per sample. Raw FASTQ files were imported into Cell Ranger 9.0 for alignment to reference transcriptome, and report of UMIs/reads/nuclei barcodes. h5 files were exported and imported into Python environment, where ambient RNA correction tool CellBender was used [145]. Processed files were imported into Seurat v5 [146], and data quality control was performed by removing nuclei with <200 UMIs, nuclei with >5% of mitochondrial reads, and genes expressed in <3 nuclei. Datasets were normalized using the SCTransform() command. Dimensionality reduction was performed using the RunPCA(), RunUMAP(), FindNeighbors(), FindClusters() commands. Doublet removal was performed using DoubletFinder(). All objects were then integrated using SelectIntegrationFeatures() from the top 3000 variable features and PreSCTIntegration(). Integration anchors were generated using the FindIntegrationAnchors() command, inputting the identified integration features to the “anchor.features” parameter, and specifying the “normalization.method” parameter as “SCT”. Datasets were then integrated by supplying the anchors to the IntegrateData() command, specifying the “normalization.method” parameter as “SCT”. A final dimensionality reduction was performed using RunPCA(), FindNeighbors(), FindClusters(), RunUMAP() with number of PCs set to 30 and resolution of 0.5. Data were then normalized and scaled with JoinLayers(), NormalizedData(), and ScaleData(). Clusters were visualized with DimPlot(). Gene expression visualization of clusters was performed using DoHeatmap(). Feature plots were generated using the FeaturePlot() function. DotPlots were created using the DotPlot() function. Violin plots were created using the VlnPlot() command. Input features for Heatmaps and Dotplots were either manually selected, or generated through the FindAllMarkers() or FindMarkers() functions.

### 4.11 Statistical Analyses

For RT‐qPCR experiments, the 2ˆ‐(∆∆Ct) method was used to calculate relative expression and fold change. Groups were compared by two‐way ANOVA test followed by a Tukey post‐hoc test. For bulk RNA‐Sequencing analyses, alignment was performed using STAR 2.7.8a, quantified to annotation model mm39, filtered for features with a maximum of <5 counts, then normalization and statistical comparisons were performed with DESeq2. Genes with a false discovery rate (FDR, Benjamini–Hochberg method) adjusted *p*‐value < 0.05 were identified as differentially expressed genes (DEGs). No fold‐change cut offs were used. For digital deconvolution of cell proportion, a two‐way ANOVA test was performed for each predicted cell‐type to determine main effects of age, MOV, or interactions. For all of the above analyses, sample sizes were n = 6–7 for each condition: adult sham, adult MOV, aged sham, aged MOV. For myonuclear RRBS analyses, differentially methylated sites were defined as q‐value of <0.05 and >10% methylation difference. During the initial stage of analysis, we found a few samples with extreme deviation from experimental conditions and potentially skewing statistical analyses. We confirmed this was not due to technical errors in tissue process or labeling. We ran a series of diagnostics on the annotated promoter matrices to assess whether any samples should be considered for exclusion. This included: 1) PCA with group centroid overlays, to visualize variance and clustering structure, 2) Centroid distance calculations, to quantify how far each sample is from the center of its group, 3) Multivariate dispersion testing, to assess within‐group spread in an unsupervised way. Based on these results, we identified five samples that clearly fell outside the expected distribution across multiple comparisons, showing extreme distances from their respective group centroids, visually separated from their cohorts, and in some cases clustered with the other experimental condition (e.g. MOV vs Sham). Given the magnitude of variance in these samples and the uncertainty of how this variance arose, we excluded these samples from analysis. This left us with samples sizes for each condition of: adult sham (n = 3), adult MOV (n = 4), aged sham (n = 3), aged MOV (n = 3). For exploratory smnRNA‐seq analyses, we had one (n = 1) biological sample for each experimental condition, which was derived from the same tissue used for the other aforementioned analyses. Differentially expressed genes between clusters or experimental conditions were identified using the FindAllMarkers() and/or FindMarkers() functions, which uses a Wilcoxon Rank Sum Test, with min.pct = 0.25, Log_2_FC > 0.25, with adjusted *p*‐value (FDR) < 0.05.

## Author Contributions

P.J.K. and K.A.M. conceived the study. P.J.K., R.G.J., A.R.C., F.M., and A.I. performed experiments and/or analysis. K.A.M, N.P.G., and J.J.M provided resources. P.J.K and K.A.M wrote the manuscript draft with input from A.I., J.J.M., N.P.G., and Y.W. All authors reviewed and approved the manuscript.

## Funding

This study was supported by NIH grants AG063944, AG080047, and AG088465 to K.A.M. This work was performed while KAM was a Glenn Foundation for Medical Research/American Federation for Aging Research Junior Investigator Awardee. Pilot work for the smnRNA‐seq experiment was funded by AR INBRE (P20GM103429) to PJK. This work was also supported by the Arkansas Integrative Metabolic Research Center (AIMRC) Center of Biomedical Research Excellence (COBRE, P20GM139768) and funds from Kate Mamiseishvili, PhD, Dean of the University of Arkansas College of Education and Health Professions.

## Conflicts of Interest

Y.W. is the founder of MyoAnalytics LLC. The remaining authors have no other competing interests to declare.

## Ethics Statement

All animal procedures were approved by the Institutional Animal Care and Use Committee of the University of Arkansas.

## Consent

The authors have nothing to report.

## Supporting information




**Supporting File 1**: advs74492‐sup‐0001‐SuppMat.pdf.

**Supporting File 2**: advs74492‐sup‐0002‐SuppMat.pdf.

**Supporting File 3**: advs74492‐sup‐0003‐SuppMat.pdf.

**Supporting File 4**: advs74492‐sup‐0004‐SuppMat.pdf.

**Supporting File 5**: advs74492‐sup‐0005‐Data.zip.

## Data Availability

The data that support the findings of this study are available in the supplementary material of this article. Bulk RNA‐seq, RRBS, and smnRNA‐seq data are deposited in the Gene Expression Omnibus (GEO) database: GSE319289. Previously published bulk myonuclear RNA‐seq data are available in GSE21340649. [Correction added on 8 April 2026 after online publication: Data Availability Statement is updated.]
