# Supplementary figures and images for: The Age‐Dependent Resident Myonuclear Multi‐Omic Response to an Acute Skeletal Muscle Hypertrophic Stimulus in Mice
Source: Adv Sci (Weinh). 2026 Feb 17;13(25):e21633. doi: 10.1002/advs.202521633 (PMC13137840; doi:10.1002/advs.202521633)

# Supplemental Figure 1

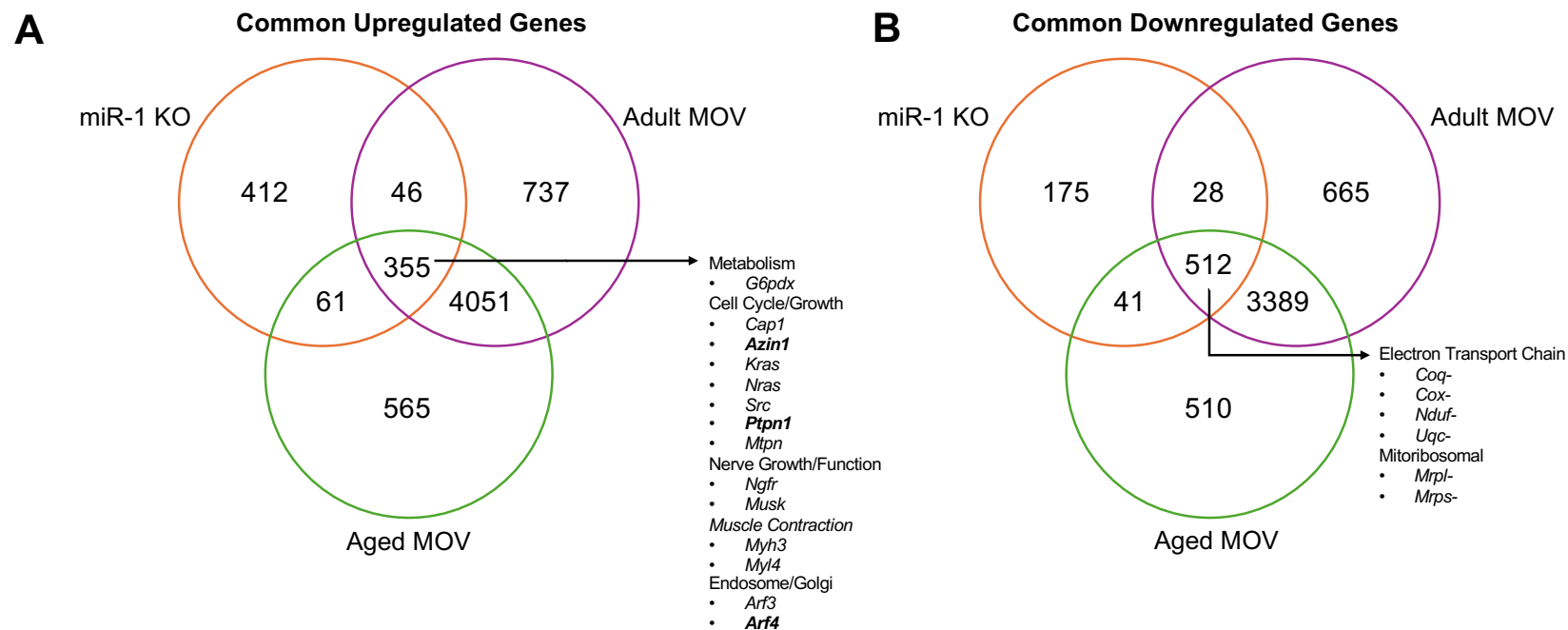

**C**

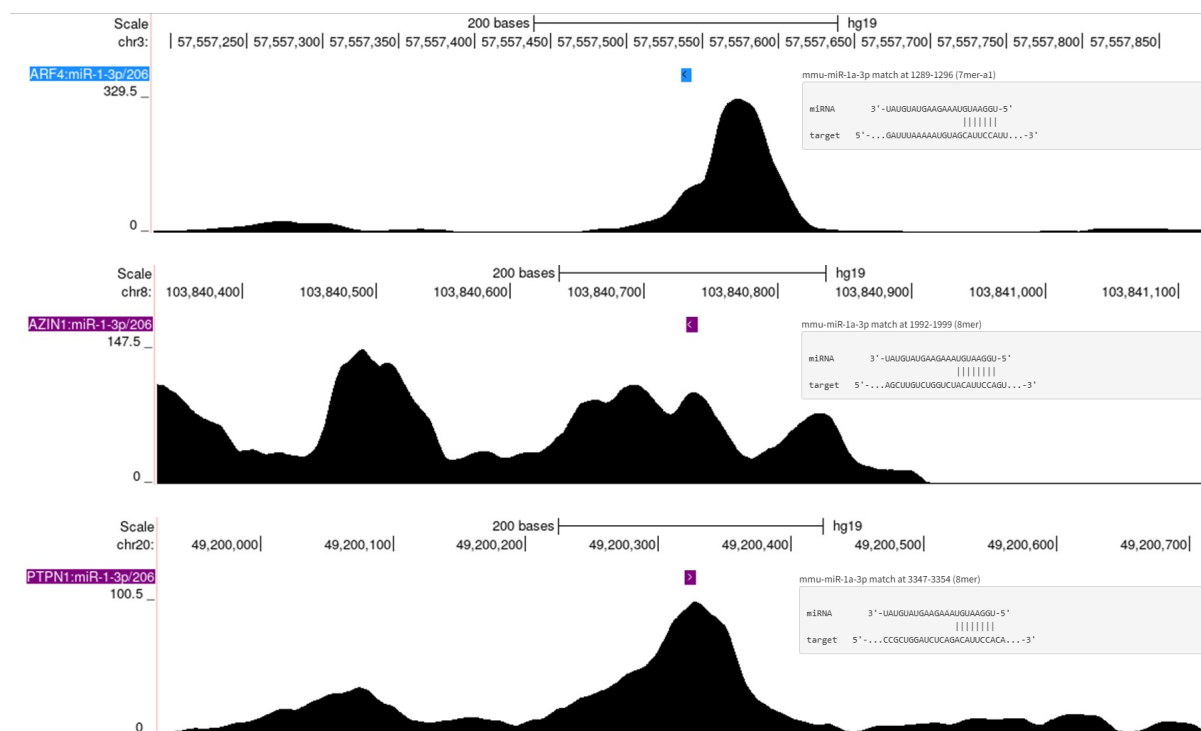

Supplement: Supplementary file 1 — Supporting File 1: advs74492‐sup‐0001‐SuppMat.pdf. [file ADVS-13-e21633-s004.pdf]

A

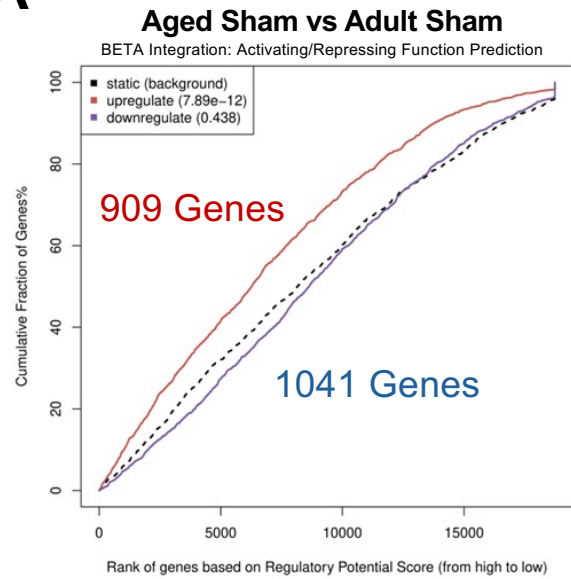

B

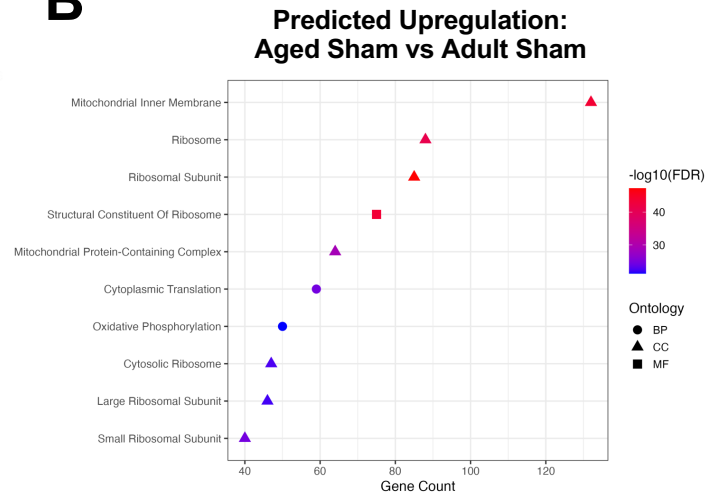

C

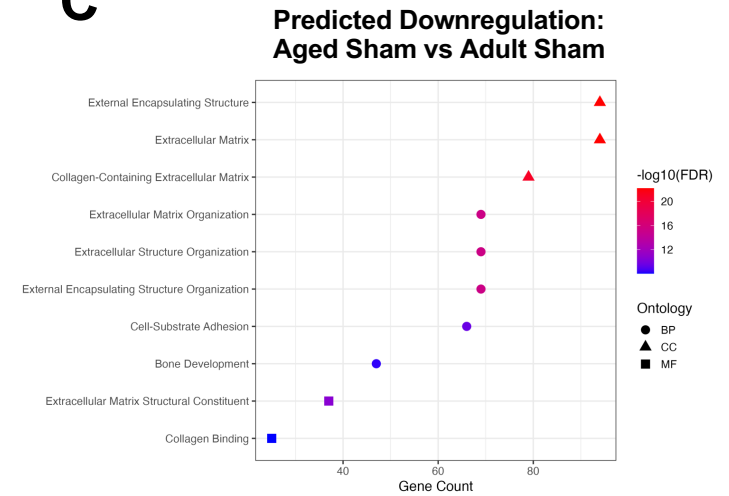

Supplement: Supplementary file 2 — Supporting File 2: advs74492‐sup‐0002‐SuppMat.pdf. [file ADVS-13-e21633-s002.pdf]

**A**

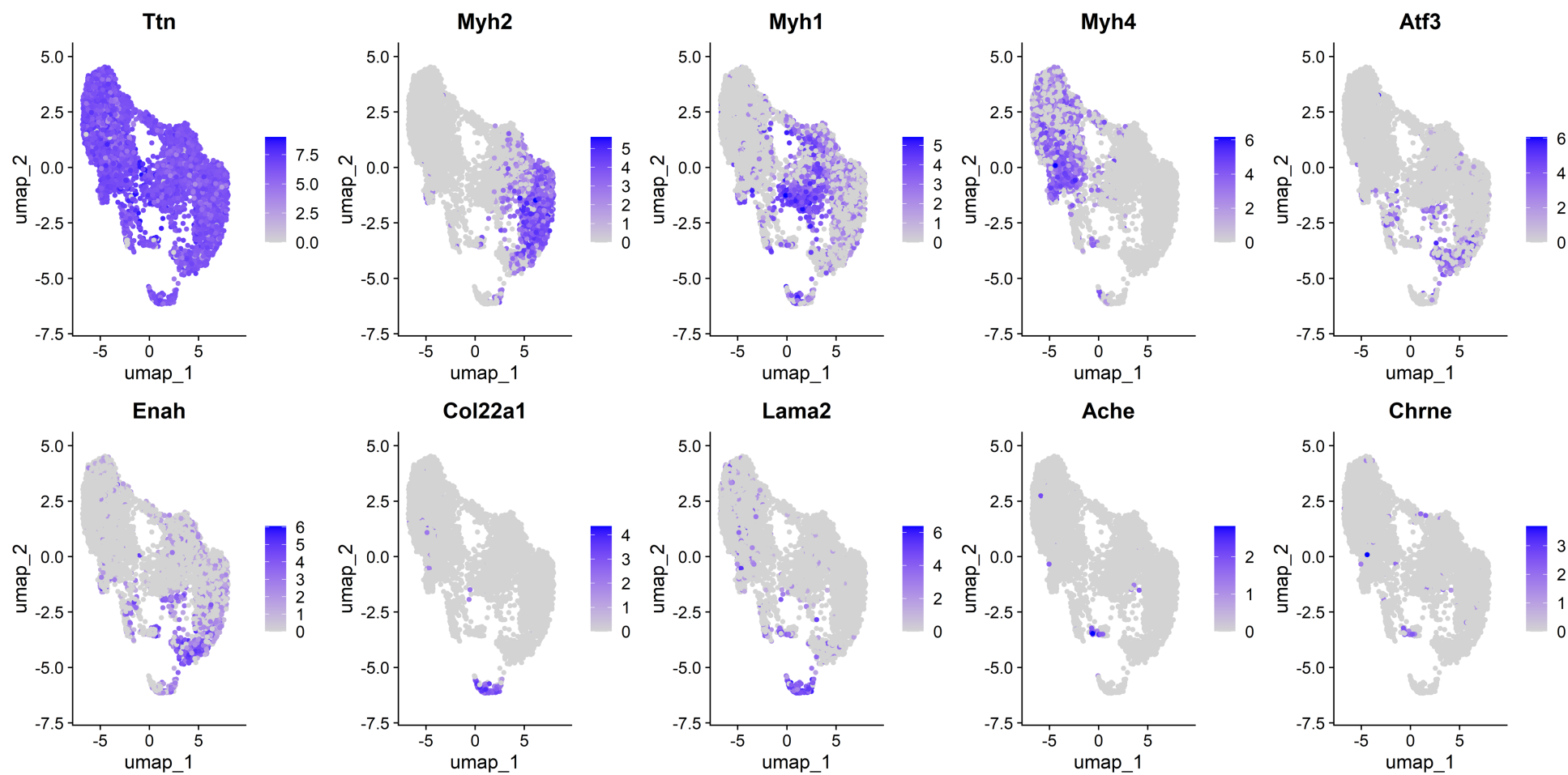

Supplement: Supplementary file 4 — Supporting File 4: advs74492‐sup‐0004‐SuppMat.pdf. [file ADVS-13-e21633-s005.pdf]
