# Supplementary material for: The Age‐Dependent Resident Myonuclear Multi‐Omic Response to an Acute Skeletal Muscle Hypertrophic Stimulus in Mice
Source: Adv Sci (Weinh). 2026 Feb 17;13(25):e21633. doi: 10.1002/advs.202521633 (PMC13137840; doi:10.1002/advs.202521633)
Supplement: Supplementary file 3 — Supporting File 3: advs74492‐sup‐0003‐SuppMat.pdf. [file ADVS-13-e21633-s001.pdf]

A

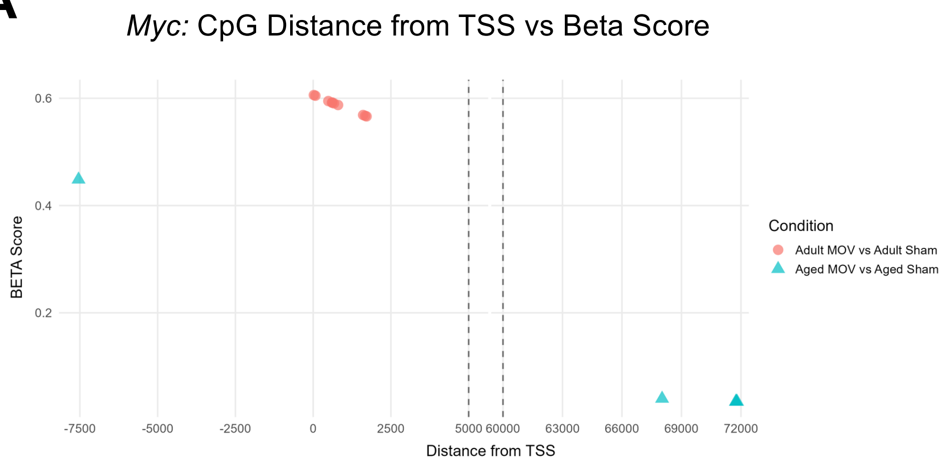

B

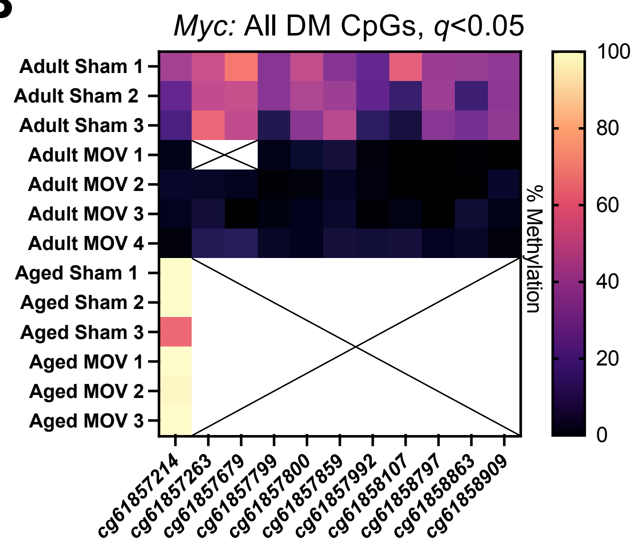

C

Gene	Adult MOV: # BETA Reg. CpG Sites	Adult MOV: Avg. Dist. from TSS	Aged MOV: # BETA Reg. CpG Sites	Aged MOV: Avg. Dist. from TSS
Ankrd1	11	48038	1	92080
Atf3	36	24567	7	53192
Runx1	19	11309	17	22361
Enah	21	7516	4	72183
Mybph	13	55646	0	NA
Myc	10	827	5	58176
Igf2bp2	22	3052	6	26020
